# Room‐Temperature Multiferroic Liquids: Ferroelectric and Ferromagnetic Order in a Hybrid Nanoparticle–Liquid Crystal System

**DOI:** 10.1002/adma.202508406

**Published:** 2025-07-26

**Authors:** Hajnalka Nádasi, Peter Medle Rupnik, Melvin Küster, Alexander Jarosik, Rachel Tuffin, Matthias Bremer, Melanie Klasen‐Memmer, Darja Lisjak, Nerea Sebastián, Alenka Mertelj, Frank Ludwig, Alexey Eremin

**Affiliations:** ^1^ Institute of Physics Otto von Guericke University Universitätsplatz 2 39106 Magdeburg Germany; ^2^ Jožef Stefan Institute Jamova cesta 39 Ljubljana SL‐1000 Slovenia; ^3^ Faculty of Mathematics and Physics University of Ljubljana Jadranska ulica 19 Ljubljana SL‐100 Slovenia; ^4^ Institute of Electrical Measurement Science and Fundamental Electrical Engineering and Laboratory for Emerging Nanometrology TU Braunschweig Hans‐Sommer‐Str. 66 38106 Braunschweig Germany; ^5^ Merck Electronics KGaA Frankfurter Strasse 250 64293 Darmstadt Germany

**Keywords:** complex fluids, multiferroics, nanomaterials, nonlinear optics

## Abstract

Responsiveness to multiple stimuli and adaptivity are paramount for designing smart multifunctional materials. In soft, partially ordered systems, these features can often be achieved via self‐assembly, allowing for the combination of diverse components in a complex nanostructured material. Here, an example of a liquid is demonstrated that simultaneously displays both ferroelectric and ferromagnetic types of order. This material is a nanostructured liquid crystalline hybrid comprising ferrimagnetic barium hexaferrite nanoplatelets suspended in a ferroelectric nematic host. Director‐mediated interactions drive the self‐assembly of nanoplatelets in an intricate network. Due to the coupling between the polar electric and magnetic types of order, this material demonstrates magnetically driven electric and nonlinear optical responses, as well as electrically driven magnetic response. Such multiferroic liquids are highly promising for applications in energy harvesting, nonlinear optics, and sensors.

## Graphical abstract

A hybrid fluid combining ferrimagnetic nanoplatelets and a ferroelectric nematic displays coexisting magnetization and polarisation at room temperature. This self‐assembled multiferroic shows strong magnetoelectric effect and nonlinear optical response, offering a soft, reconfigurable platform for next‐generation sensing, actuation, and photonic applications.

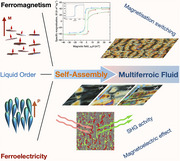

## 1 Introduction

Soft nanostructured materials such as colloids and liquid crystals (LC) have become very interesting for self‐assembly behavior and the ability to control their properties using weak external stimuli.^[^ ^1^ ^]^ These materials have a wide range of applications, particularly in display, optical and communication technologies. In addition, they hold significant promise for wearable electronics and healthcare.

**Figure 1:**
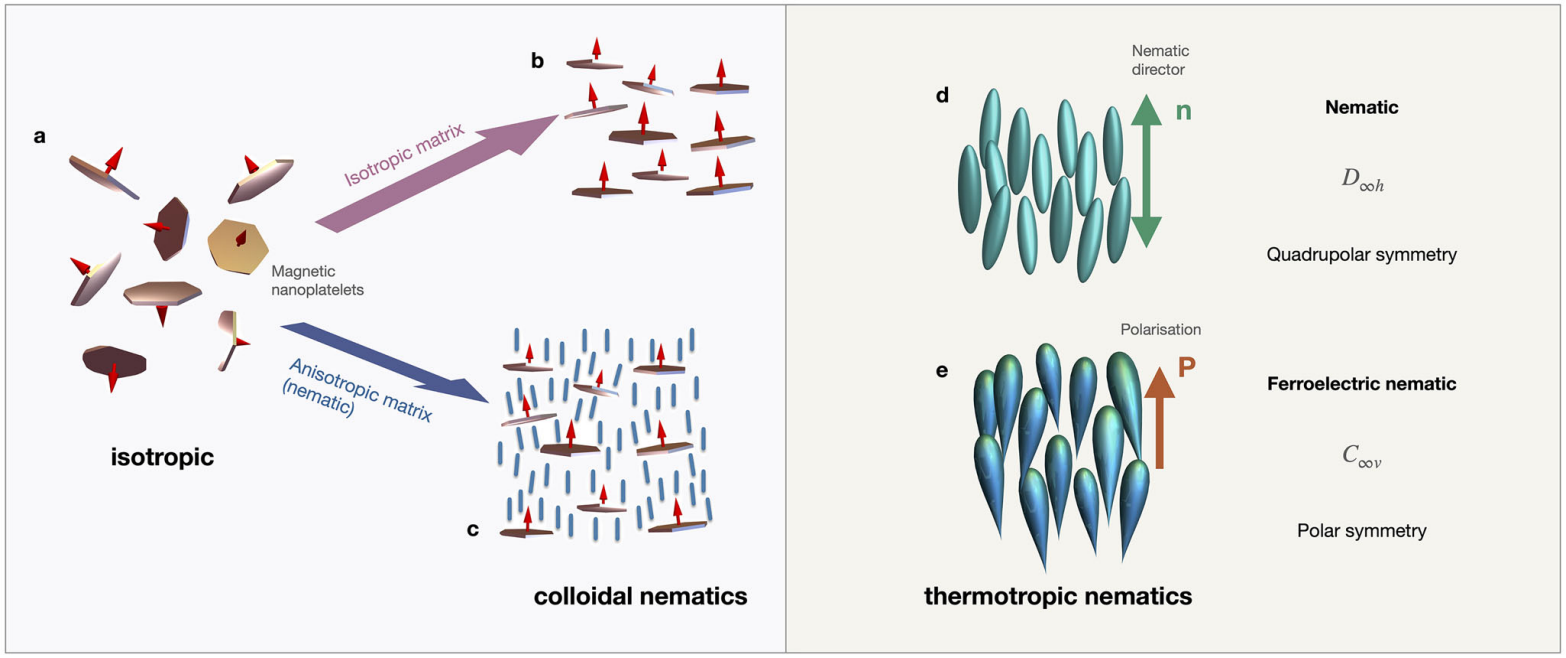
Fluids with orientational order: a) isotropic suspensions of BaHF nanoplatelets in 1‐butanol form b) the colloidal ferromagnetic nematic phase at a sufficiently high volume fraction. c) Ferromagnetic nematic phase occurs in dispersions of the nanoplatelets in a thermotropic nematic. d) Thermotropic nematogens can form the nematic phase N with a cylindric symmetry. e) In some compounds, the director invariance is broken, resulting in the ferroelectric nematic phase N_F_ with the director's polar symmetry.

In solids, intermolecular interactions stabilize ordered crystalline structures, giving rise to materials with various types of order. Some of them can even be ferromagnetic or ferroelectric. In contrast, in liquids, such as liquid crystals and colloids, it is a delicate balance between interactions and entropy accompanied by symmetry‐breaking instabilities stabilizing different kinds of order.^[^ ^2^, ^3^ ^]^ Conventional nematics, widely used in modern display technology, have up‐down (quadrupolar) symmetry and do not exhibit any spontaneous polarization.^[^ ^4^, ^5^ ^]^ Recent years, however, were marked by two important discoveries in the field of liquid crystals: A liquid ferromagnet was discovered in the suspension of scandium‐doped barium hexaferrite (BaHF) nanoplatelets (**Figure** **1**a–c),^[^ ^6^, ^7^, ^8^ ^]^ and a true 3D ferroelectric liquid, a ferroelectric nematic (Figure 1d,e), was found to be formed by strongly polar mesogens.^[^ ^9^, ^10^, ^11^, ^12^, ^13^, ^14^ ^]^ In contrast to conventional nematics with axial symmetry, these fluids are distinguished by their vector‐type properties, such as spontaneous electric polarization in the ferroelectric nematic N_F_, and spontaneous magnetization in the ferromagnetic nematic N_M_. The magnetic structure in N_M_ is determined by the self‐assembly of the ferrimagnetic nanoplatelets dispersed either in a nematic host or in an isotropic liquid.

In the first case, the director‐mediated interactions stabilize the order of the nanoplatelets.^[^ ^6^, ^7^, ^8^ ^]^ The elastic energy of the director deformation in the bulk is coupled to the positional and orientational degrees of order of nanoplatelets (or other inclusions) through the surface anchoring, resulting in anisotropic interactions between them.^[^ ^15^, ^16^, ^17^, ^18^ ^]^

In the latter case, a colloidal nematic is stabilized by steric, electrostatic and magnetic interactions. Ferroelectric order in nematics was predicted in the early twentieth century but discovered only recently.^[^ ^9^, ^19^, ^20^, ^21^ ^]^ The actual stabilization mechanism is still under intensive investigation. N_F_ materials are distinguished by their unusually high spontaneous polarization (up to 10 µC/cm^2^), comparable to solid ferroelectrics, and high optical nonlinearity. The latter makes those materials promising for applications in photonics, such as frequency converters and modulators. The fluidity in these hyperpolar materials brings about new extraordinary properties such as super‐screening, mechanoelectrical effect, stabilization of fluid fibres, and electrohydrodynamic instabilities.^[^ ^22^, ^23^, ^24^, ^25^, ^26^, ^27^, ^28^ ^]^

Multiferroics are materials exhibiting multiple ferroic orders simultaneously–most commonly ferroelectric or ferroelastic and ferromagnetic properties.^[^ ^29^, ^30^, ^31^ ^]^ Notable examples include BiFeO_3_ and EuTiO_3_. These materials have become technologically significant due to their coupled magnetoelectric properties, enabling the control of magnetic states by electric fields and vice versa. Such capabilities are central to applications ranging from highly energy‐efficient actuators and sensors to non‐volatile data storage and energy harvesting.^[^ ^32^, ^33^, ^34^, ^35^, ^36^, ^37^, ^38^ ^]^ Like classical ferroelectrics, multiferroics can serve as model systems for artificially constructing, and reversibly switching solid–liquid interfacial states in fluid environments, a capability particularly beneficial for advanced surface‐chemistry applications.^[^ ^39^, ^40^, ^41^ ^]^ However, designing solid multiferroics remains challenging because of conflicting chemical requirements: ferroelectricity favors transition metals with empty *d* orbitals, whereas ferromagnetism prefers partially filled *d* orbitals.^[^ ^30^ ^]^ Various strategies, including the development of composite materials, have successfully addressed this issue.^[^ ^30^, ^42^ ^]^

An attractive alternative approach involves liquid multiferroics, which combine the deformability and self‐healing properties of soft matter with the multifunctionality and responsiveness characteristic of their solid‐state counterparts. The primary advantage of liquid‐phase multiferroics lies in their extreme sensitivity to external stimuli and their optical properties. For example, ferromagnetic nematic suspensions exhibit giant magneto‐optical response at remarkably low magnetic fields of just a few millitesla, facilitating contact‐free visualization and mapping of magnetic fields.^[^ ^43^ ^]^ One can envision that, introducing a coupling between magnetic and polar electric orders in such systems would add an independent electrical channel for tuning material responses, further enhancing versatility. Because these fluids would easily form functionalizable films and emulsions, they are particularly promising for applications in surface chemistry and interfacial sensing, especially for designing voltage‐programmable catalytic interfaces, whose activity can be spatially rewritten on demand. In contrast, reversible elastic switching in solid multiferroics, even in thin films, is often impeded by substrate clamping, requiring elaborate micro‐ or nanopatterning, or the development of intrinsic orthorhombic heterostructures, as demonstrated by Wang et al.^[^ ^39^ ^]^

In this paper, we demonstrate the first liquid multiferroic composed of ferrimagnetic BaHF nanoplatelets dispersed in a ferroelectric nematic liquid crystal. The suspension forms a hybrid liquid crystal (hybrid‐LC) material which remains stable in a wide range of temperatures, including room temperature. In addition to the electro‐ and magneto‐optical responses, this material exhibits direct and converse magnetoelectric responses.

## 2 Results

### 2.1 Structure and Morphology Characterization

Barium hexaferrite magnetic nanoplatelets have profound effect on the structure and morphology of the multicomponent ferroelectric nematic. The pure liquid crystal exhibits the high‐temperature nematic (N) phase, the intermediate antiferroelectric (M) phase^[^ ^44^ ^]^ and the low‐temperature ferroelectric nematic (N_F_) phase with transitions given below:

(crystal < −20 °C) N_F_ 45.8 °C M 57.9 °C N 87.6 °C isotropic

The nanoplatelets were suspended in the LC host with concentrations ranging from 0.3 wt% to 4.0 wt% followed by quenching from the isotropic phase to the N_F_ in a magnetic field µ_0_ *H* = 482 mT. The suspensions did not significantly alter the transition temperatures at this concentration range.

One of the most remarkable features of the nanoplatelet/N_F_ hybrids is the multifaceted response to external electric and magnetic fields. This behavior was characterized in 2.5 µm − 30.0 µm glass planar cells filled with the hybrid liquid crystal. Transparent indium tin oxide (ITO) electrodes on the cell substrates allow applying electric field and measuring the material's response simultaneously. The hybrid material exhibits, similarly to the pure N_F_ phase, the ferroelectric‐type current transients on polarity reversal (Figure S1, Supporting Information) and SHG activity (Figure 3). Additionally, it shows remanent magnetization in the field‐free state (Figures 3a; Figure S2, Supporting Information).

**Figure 2:**
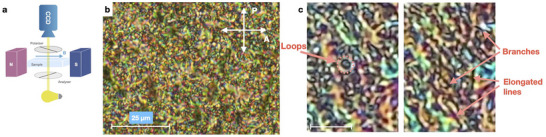
Optical morphologies in the hybrid‐LC: a) Schematics of the polarizing microscopy setup for optical textures characterization in a magnetic field. b) Polarizing microscopy textures observed in hybrid‐LC in 10 µm cell with polyimide rubbed substrates in the N_F_ phase at *T* = 34 °C. c) Various topologies of disclination lines in a 5 µm cell without aligning layer. The white line corresponds to 7 µm.

**Figure 3:**
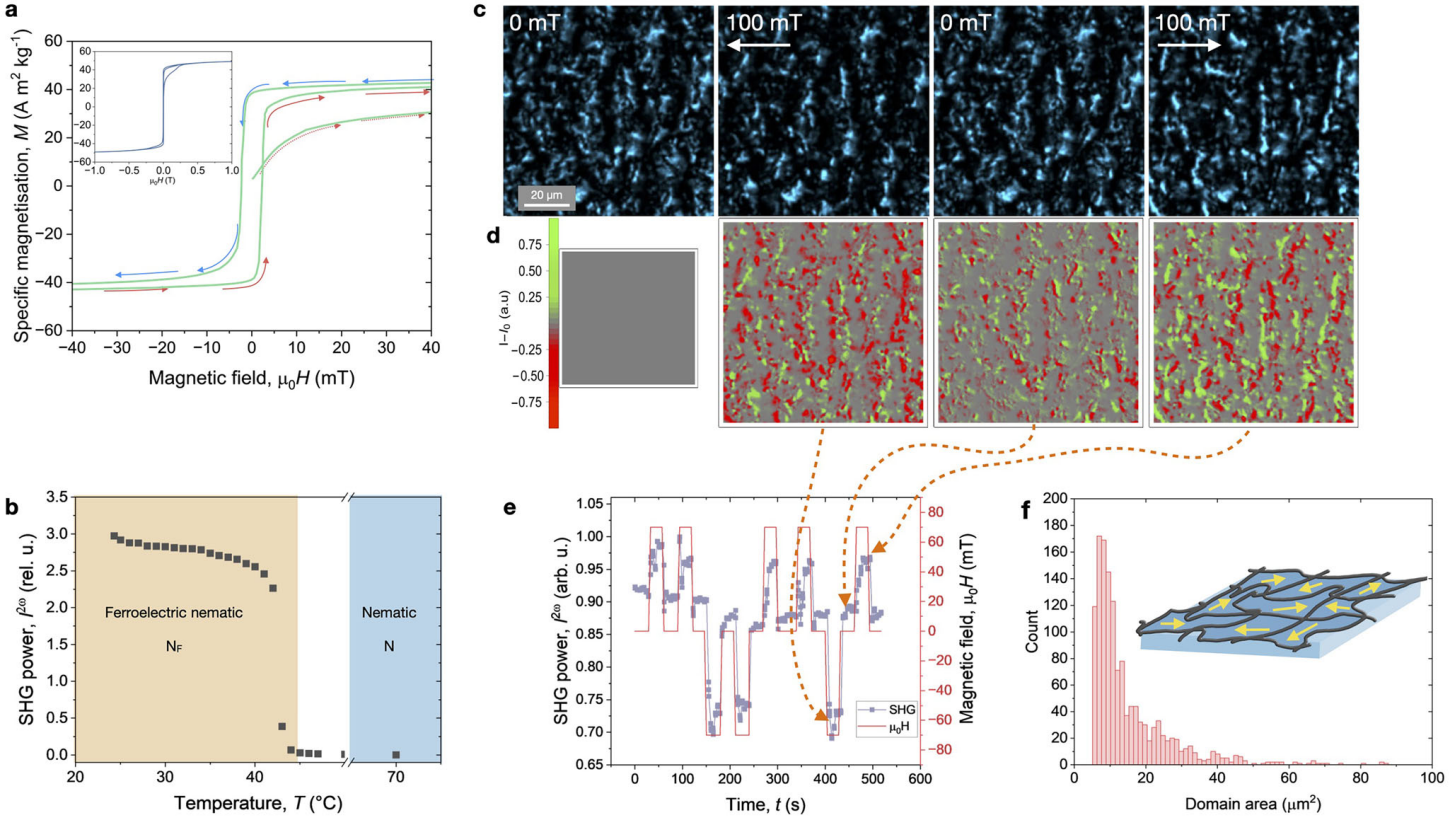
Magnetic and polar characterization of the hybrid‐LC: a) Hysteresis loop of specific magnetization *M*(*H*) measured in a 1 wt% hybrid‐LC within a glass capillary, revealing characteristics of ferromagnetic switching. Inset provides the complete field range. b) Variation of spontaneous SHG signal with temperature in a planar‐aligned 5 µm cell. c) Sequence of SHG microscopy images acquired from a 5 µm‐thick planar‐aligned cell subjected to an external magnetic field (field values indicated on each frame). The full time‐lapse is provided in Movie S3 (Supporting Information). d) Corresponding difference images obtained by subtracting the initial field‐free frame from each subsequent frame shown in (f), highlighting SHG signal changes induced by the field. e) Time plot of the mean SHG intensity, averaged over a 100 µm^2^ region of the sample under repeated magnetic field exposure. Orange arrows correspond to selected frames in c/d, marking key time points on the intensity trace. f) A schematic of a polydomain structure of the sample with the arrows indicating the local spontaneous magnetic order and a distribution of the domains' areas in the sample.

**Figure 4:**
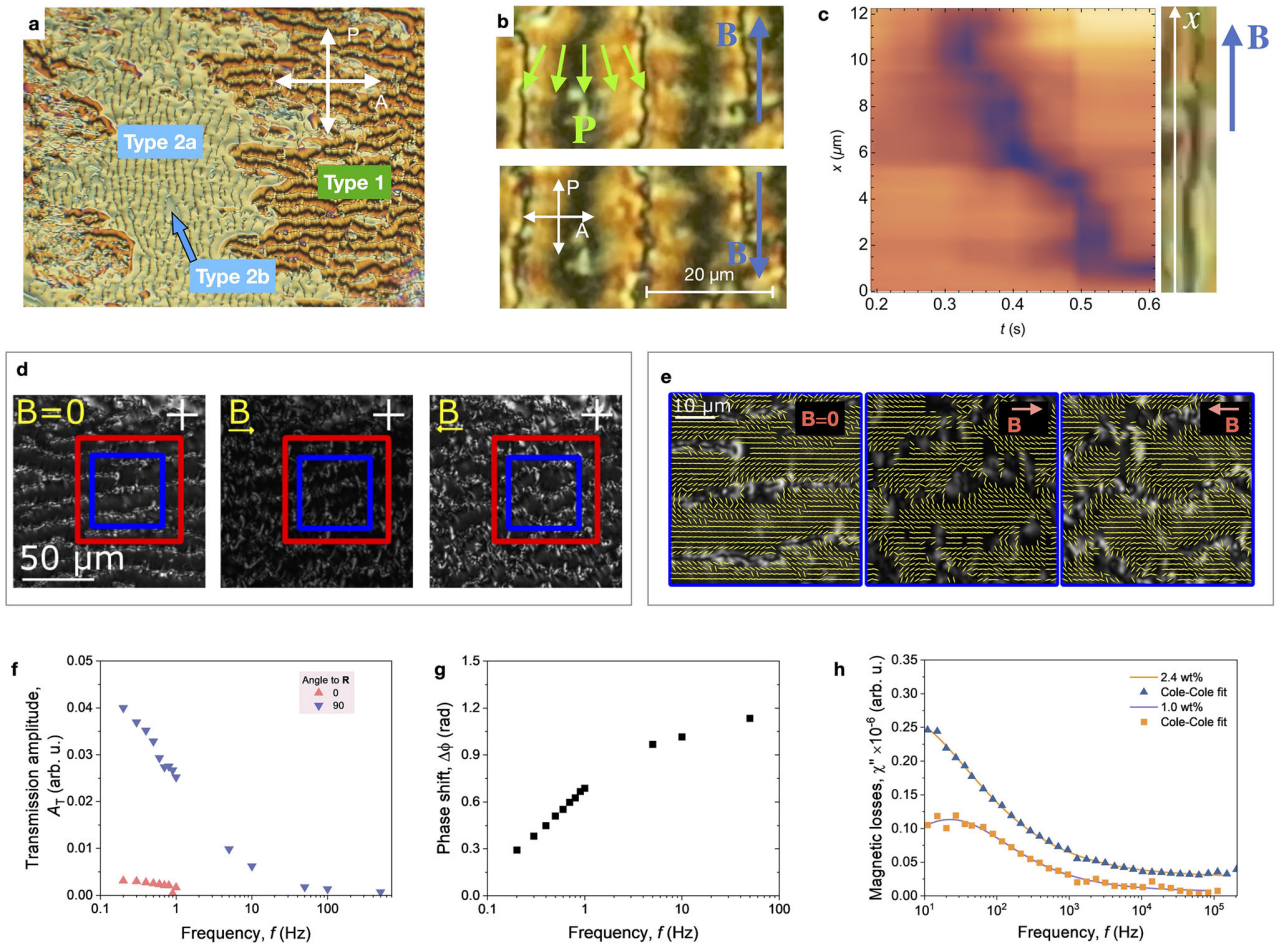
Magneto‐optical behavior in thin cells a) POM image of the N_F_‐hybrid texture in a 2.5 µm P‐type cell between crossed polarizers. b) Microscopic texture of magnetically prealigned sample in a 2.5 µm thick glass cell with rubbed polyimide aligning layer. The green arrows indicate the spontaneous polarization as inferred from the SHG characterization. c) Space‐time plot of the mean transmission measured parallel to a disclination line (displayed on the right) under application of *B* ≈ 140 mT (Movie S6, Supporting Information). d) POM images correspond to the cases without a magnetic field and with applied magnetic fields of opposite polarities. e) The polarization field extracted from the SHG microscopy marked with yellow lines is overlain with the POM images marked blue in (d). f) Frequency dependence of the transmission amplitude for the field **B** applied parallel (0°) and perpendicular (90°) to the rubbing direction **R**. g) Frequency dependence of the phase shift Δϕ between the phase of the AC magnetic field and the phase of the induced optical response (transmission) to the field aligned perpendicular to **R**. (Cell thickness: 3 µm, field amplitude: 5 mT, measurement area 1 mm^2^ (see Note S4, Supporting Information)). h) Spectra of magnetic losses χ″(*f*) of nanoplatelet/N_F_ hybrid recorded at room temperature for two nanoplatelet concentrations, 1.0 wt% and 2.4 wt%.

Optical polarizing microscopy (POM, **Figure** **2**a) provides a tool to explore the molecular order and alignment on micro‐ and macroscopic scales (Note S3 and Figures S3 and S4, Supporting Information). In the N_F_ phase, the morphology of the hybrid material, as revealed by POM, primarily depends on the cell thickness and the surface treatment (Figures 2 and **4**a,b; Note S4 and Figures S5 and S6, Supporting Information). Cells with rubbed polyimide treatment and surfactant cetyltrimethylammonium bromide surface layers were used for planar (P‐cells) and vertical (V‐cells) alignments in the nematic, respectively.

In both cell types, irregular textures strongly depend on the filling conditions. Diverse morphologies were found in different regions across the same cell, as described in Note S4 and Figures S5 and S6 (Supporting Information). Interestingly, in some areas of V‐cells, a response to the magnetic field was observed in small uniform patches (Figure S5d,e, Supporting Information).

In thick (*d* ⩾ 5 µm) cells, the most common birefringent optical textures exhibited a disordered, grainy morphology responsive to electric and magnetic fields (Figure 2b; Figure S3 and Movies S1 and S2, Supporting Information). Close inspection of the POM textures revealed a disordered grainy texture, indicating that the structure of this phase consists of a complex network of entangled lines (Figure 2b,c). Although the precise structure of these lines remains unresolved at this stage, they are commonly interpreted as topological line defects (disclinations) or inversion walls, characteristic of nematic and ferroelectric nematic phases. A clearer picture of the lines is obtained in thin cells, where the single lines can be well resolved (Figure 4). This morphology was observed in thick layers of all studied suspensions, irrespective of the anchoring conditions.

The textural changes in response to external fields are particularly evident in cells with a thickness of 5 µm. Despite being highly disordered, the network of threads readily rearranged in response to a magnetic field **B** (Movie S2, Supporting Information). The material's ferromagnetic nature became evident upon repeated stimulation by **B**. When the field was removed, the established texture only partially relaxed. Reapplying a field with the same polarity caused only minor changes in the morphology. However, when a field of the opposite polarity was applied, the texture underwent complete reassembly, indicating that the ground state of the hybrid N_F_ phase was ferromagnetic (Movie S1, Supporting Information). The remanent magnetization of the cells was demonstrated by direct measurements of the magnetization using a fluxgate probe (Figure S2, Supporting Information).

The measurements of the magnetization in thin 1 mm capillaries using SQUID magnetometer confirmed hysteresis behavior of the magnetization (**Figure** **3**a). The specific magnetization per unit mass of the magnetic component in the hybrid reaches saturation at approx. 40 Am^2^/kg, which is in good agreement with the values obtained from powder samples of BaHF platelets.^[^ ^45^ ^]^

Two experimental techniques allow probing the polar structure of mesophases: measurements of the current transients on polarization reversal and detection of the nonlinear optical second harmonic generation (SHG). Noncentrosymmetric structures of a ferroelectric phase with *C*_∞*v*_ symmetry generate non‐zero components of the second‐order hyperpolarizability tensor, allowing for SHG. In the phases with a centrosymmetric structure, such as the nonpolar nematic phase, SHG is forbidden.

In the non‐doped LC, the ferroelectric N_F_ phase has a large spontaneous polarization aligned along the nematic director.^[^ ^25^ ^]^ The hybrid‐LC shows a similar SHG activity in the temperature range of the N_F_ phase. The SHG signal *I* ^2ω^(*T*) of a sample cell slightly decreases as the temperature increases, as shown in Figure 3b. Only when the transition to the M phase occurs does the signal drop to the background level, indicating that the M phase is not SHG active. To gain a better understanding of the polar behavior, we utilized SHG microscopy.

Figure 3c,d display the SHG microscopy images of a 5 µm thick nanoplatelet/N_F_ cell. The field‐free state showcases a very disordered texture, consisting of grains with different orientations of the polar domains with typical average areas of 15 µm^2^ and standard deviation of σ = 12 µm^2^ (Figure 3f). In a magnetic field, *I* ^2ω^ increases, indicating local alignment of the polar axes. Similarly to the optical textures, removing the magnets causes partial relaxation. However, if the field is repeatedly applied with the same polarity, the response diminishes, indicating that the magnetic state of the sample is prealigned. Reversing the magnetic field causes a significant reorganization of the network, leading to changes in the *I* ^2ω^ signal (Figure 3e). This nonlinear optical behavior reflects the coupling between magnetization and electric polarization, demonstrating the magnetoelectric properties of the hybrid material.

To suppress the formation of the disordered entangled state, we confined the liquid crystal in thin 2.5 µm cells. The POM images show large domains on a millimeter scale (**Figure** **4**a; Figure S4a,b, Supporting Information). The domains are distinguished by their optical transmittance and their behavior upon the sample rotation or insertion of a wave plate retarder.

Some domains (Type 1) display high contrast between states of transmission maxima and minima upon a 90° rotation between crossed polarizers. In contrast, Type 2a and 2b domains show no extinction, suggesting a twisted director structure with opposite handednesses (Note S3 and Figure S4c, Supporting Information). Type 1 domains exhibit a regular pattern of thin and thick lines with a quasi‐periodic structure on a scale of 20 µm at room temperature (Figure 4b). The optical transmission analysis suggests a splay deformation of the director with the alignment along the striped pattern in the middle between the thin disclination lines, corresponding to the π‐flip of the polar director. (Figures S4d–g and S7, Supporting Information). The stripes respond to an external magnetic field (Movie S4, Supporting Information). The preferred direction is determined by the magnetic field applied during sample annealing. When a magnetic field is applied along this direction, the pattern undergoes a minor rearrangement, resulting in the straightening of the disclination lines (Figure 4b top). A field in the opposite direction disturbs the disclination lines leading to a buckling instability (Figure 4b, bottom). These observations imply that the nanoplatelets are incorporated into the disclination lines, with a component of their magnetization aligned parallel to the lines. The particle's coupling to the nematic director in the volume occurs via the director anchoring at the disclinations' interface. Additionally, the particles are anchored in the disclination lines and cannot freely rotate under moderate fields. A threshold field in the range of 120 mT is required for reassembly of the lines. Such switching occurs in a soliton‐like fashion as a propagating deformation (Movie S5, Supporting Information).

As shown in Figure 4c, propagating director distortions were observed upon the application of a magnetic field of approximately 140 mT. Significant textural transformations occur upon field inversion, suggesting bistability in the system. In contrast, applying a magnetic field of the same polarity induces only minor distortions. However, strong anchoring of the director at the substrates restricts reorientation away from the disclination lines.

The polar structure of the striped pattern revealed by SHG microscopy indicates the highest SHG efficiency for the polarization direction primarily along the stripes allowing to map the local polarization field (Figure 4d,e; Note S5, Supporting Information). In agreement with the polarizing microscopy, the director exhibits a splay deformation with an oblique anchoring at the defect lines. Realignment of the defect lines by the external magnetic field deforms the director field by bending the director via anchoring thoroughgoingly at the defect lines. However, in such thin cells, most lines are firmly attached to the substrate and cannot be easily reconfigured. In this case, applying magnetic field reduces the SH signal for the primary beam polarized along the stripes.

To characterize the dynamic optical response in magnetically aligned samples, we used oscillating magnetic fields applied parallel and perpendicular to the alignment direction **R**. The samples were confined in a 3 µm cells with parallel rubbing, and a twist‐free region was selected for the measurement. The maximal field amplitude was 5 mT, which is much smaller than the field required for the magnetization reversal. Figure 4f,g shows the optical transmittance recorded between crossed polarizers. Only a small transmittance change was observed when the field was applied along the alignment direction **R**. Application of the field perpendicular to **R** results in a response following the driving field. Since the sample does not have a perfect uniform alignment but consists of distributed polydomains, the transmission is significantly higher in the field‐free state.

#### 2.1.1 Magnetically‐Induced Electric Effect

In nematics, magnetic field allows manipulation of the effective birefringence and the orientation of the optical axis. However, the magnetic field‐director coupling does not allow converting the magnetic field into an electric voltage.

**Figure 5:**
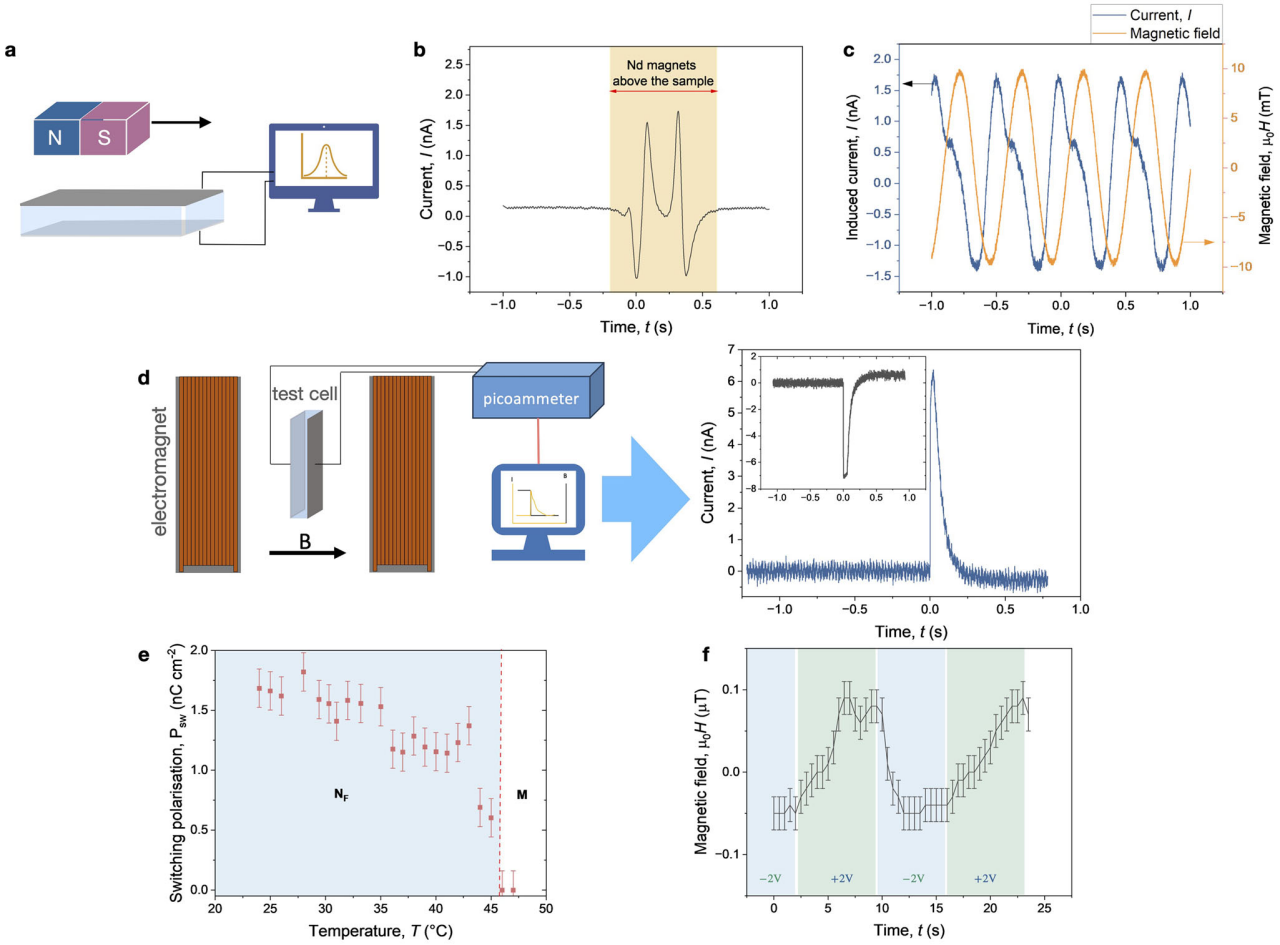
Magnetically‐induced electric and electrically‐induced magnetic effects: a) Schematic demonstration of the magnetoelectric effect: a neodymium (NdFeB) magnet passing over a sample cell induces an electric current shown in (b). c) Electric current induced in a cell placed on a magnetic stirrer which generates a rotating magnetic field (amplitude 10 mT, 2.0 Hz). d) Schematics of the measurement setup and the current transient measured in magnetically aligned samples upon inversion of the magnetic field (500 mT) in a 10 µm thick cell. The inset shows the response in the field of opposite polarity. e) Switching polarization calculated by integration of the current transient. f) Electrically‐induced magnetic response on poling the sample cell using 2 V square‐wave pulses in samples with 4 wt% nanoplatelets at room temperature measured by a fluxgate sensor on top of the cell.

In multiferroic nematic, however, the electric polarization induced by the magnetic field drives the current transient in the connected circuit. To directly demonstrate this effect, a tiny permanent magnet was hovered over a 5 µm thin layer of N_F_‐hybrid that lies between two ITO electrodes, as illustrated in **Figure** **5**a,b. The electric current generated, as shown in Figure 5b, exemplifies the fundamental principle of a basic magnetic sensor based on hybrid N_F_.

Another demonstration is given in Figure 5c, displaying the current response of an LC cell placed on a heat plate with a magnetic stirrer producing a rotating magnetic field. The magnetic field generates current at the same frequency, showing a complex but periodic behavior. This effect is observed only in the temperature range of the multiferroic nematic phase, and it disappears upon the transition into the high‐temperature M phase.

In these experiments, however, the magnetic field is not uniform. Therefore to explore the magnetically‐induced electric response, we placed the LC cell in a uniform magnetic field of an electromagnet allowing to produce magnetic fields up to µ_0_ *H* = 650 mT. The magnetically‐induced electric response to switching the magnetic field on from 0 mT to 500 mT is shown in Figure 5d. Integration of the current transient allows us determining the switching polarization *P*_sw_ whose temperature dependence is given in Figure 5e. The polarization is nearly temperature‐independent in the temperature range of the N_F_ phase and abruptly decreases upon the transition into the M phase. The switching has a distinct polar character: changing the polarity of the magnetic field results in the inversion of the current response sign and, consequently, the sign of *P*_sw_ (inset in Figure 5d).

#### 2.1.2 Converse Magnetoelectric Effect

The multiferroic character of the material assumes a converse magnetoelectric effect, where an electric field applied to the LC induces a magnetic response. To test this effect, we placed a sensitive fluxgate sensor on top of a 30 µm cell with sandwiched planar electrodes. As the samples were prepared by quenching in a magnetic field, the sample cells have a small remanent magnetization as shown in Figure S2 (Supporting Information), where the field is recorded. This magnetization can be detected by inserting the cell under the fluxgate detector, measuring the magnetic flux density at the top of the sample. Flipping the cell results in the inversion of the magnetic field sign suggesting that the cell acts as a small fluid magnet.

Application of a DC voltage (2 V) results in a magnetic response shown in Figure 5f. This response is very small but still detectable. Voltage inversion results in a reduction of the magnetization. However, the induced magnetic field decreases with time due to the loss of alignment, particle diffusion and electrically‐induced flows in the cell.

## 3 Discussion and Outlook

The behavior of the composite multiferroic nanoplatelet/N_F_ phase differs significantly from that of the separate components' ferroelectric or ferromagnetic nematic phases. Instead, the volume of the material is filled with a network of threadlike disclination lines (Figure 2b,c).

**Figure 6:**
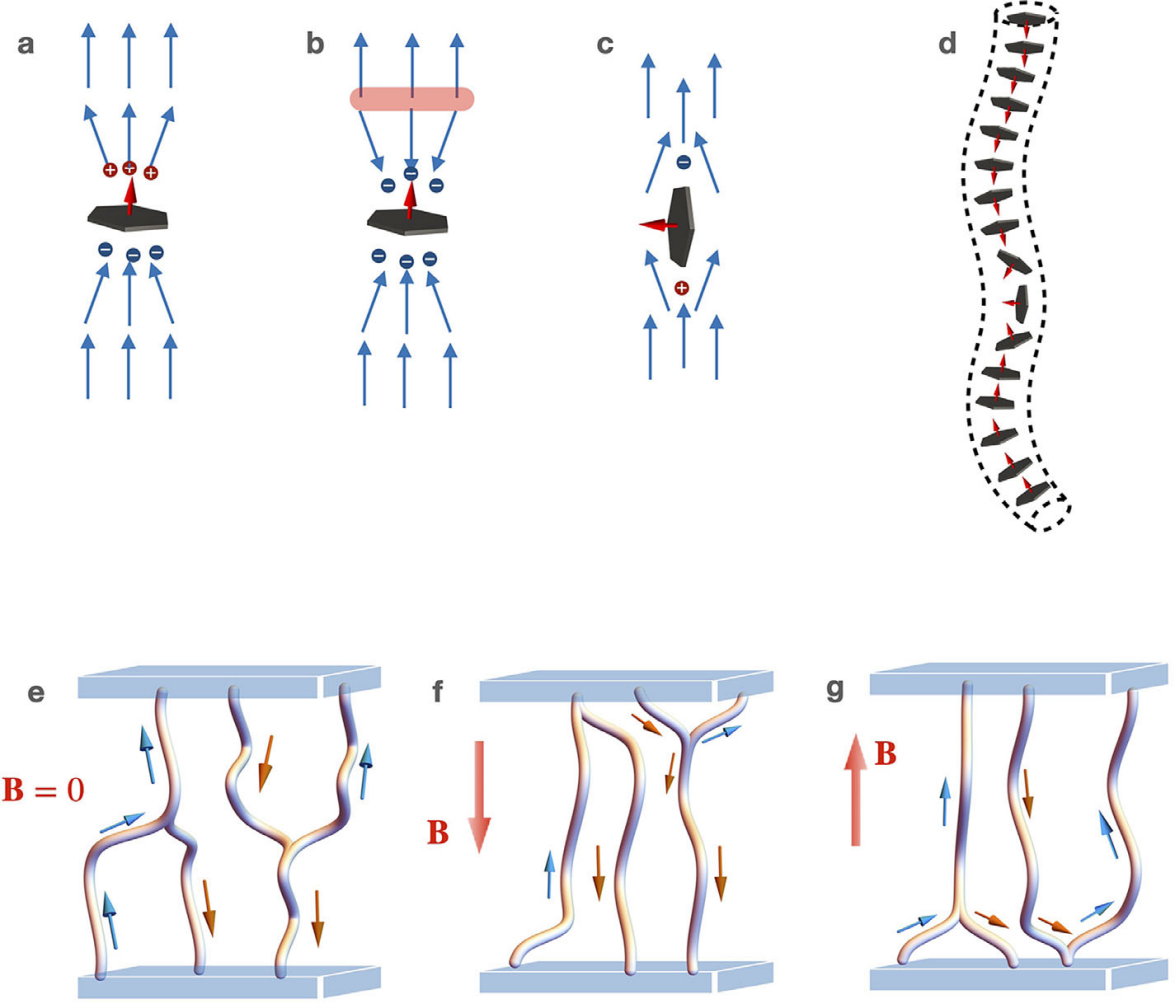
Proposed structures and network arrangements in the multiferroic N_F_ state: a) Orthogonal anchoring of the polar director (blue arrow) with continuous normal component at the nanoplatelet's surface creating opposite polarization charges. b) Discontinuous orthogonal anchoring of the polar director requires an inversion wall (marked as a pink line) to comply with the uniform director in the far field. c) Planar anchoring of the director resulting in the topological (and electric) dipole. d) A schematic model of a disclination line with self‐assembled nanoparticles forming a magnetic inversion wall. e–g) Magnetization switching scheme with an example of tripled disclination lines. The blue and orange arrows show the magnetization direction. e) In the field‐free state the disclination link in the middle provides a state without magnetization. f,g) Depending on the polarity of the applied magnetic field **B**, magnetization is established by the displacement of the link.

The observed behavior of lines in response to an external magnetic field indicates that magnetic nanoparticles are confined within them (**Figure** **6**). Meanwhile, the occasional occurrence of uniform magnetically responsive domains (Figure S6a,b, Supporting Information) suggests that nanoplatelets are also dispersed throughout the bulk material.

The dynamics of magnetic nanoparticles are affected by the strong coupling to the director and the constraints of the nematic. Even in oscillating magnetic fields as small as 5 mT, frequency‐dependent optical response can be observed (Figure 4f,g). The magnetic dynamics exhibits slow relaxation dynamics in the ACS spectra (Figure 4h). The relaxation rates can be estimated from fitting the AC susceptometry spectra with a Cole‐Cole model:

(1) $$\chi \left(f\right)=\chi_{\infty }+\frac{\Delta \chi }{1+{\left(2\pi if\tau_{B}\right)}^{1-\alpha }}$$

where χ_∞_ is the susceptibility in the high‐frequency limit, Δχ is the relaxation strength of the mode and τ_B_ is the Brownian relaxation time constant. Compared to the isotropic suspension of nanoplatelets,^[^ ^46^ ^]^ the relaxation rate is strongly reduced by more than two orders of magnitude from 700 Hz to 27 Hz, suggesting strong coupling between the LC and the nanoplatelets.

Low compatibility of the nanoplatelets with the bulk N_F_ can be explained by the frustration imposed by the polar symmetry of the director and the anchoring conditions. Uniform director alignment in ferromagnetic nematics necessitates orthogonal anchoring of the polar director at the nanoplatelet surface, which can be either asymmetric (Figure 6a) or symmetric (Figure 6b). Asymmetric anchoring preserves the continuity of the polar director across the nanoplatelet, maintaining a uniform orientation above and below. In contrast, symmetric orthogonal anchoring disrupts this symmetry, requiring the formation of complex defect structures–specifically, director inversion walls–to satisfy the uniform far‐field alignment (Figure 6b). In the latter scenario, substantial electrostatic energy penalties arise due to the emergence of localized splay domains, which induce polarization splay and the generation of bound charges ρ_b_ = −∇ · **P**. The planar anchoring would reduce the distortion energy costs, leaving two charged defects and two topological charges at the opposite sides of the platelet (Figure 6c). In both cases, a and b, the particles have a dipolar property, favoring the self‐assembly of particles in dipolar chains.

Defect lines with isotropic order within the cores may favor 1D magnetized structures of nanoplatelets like those proposed on the sketch in Figure 6d. Additional charge at the nanoplatelets' surfaces would stabilize oblique anchoring of the nematic director at the defect lines.

In the proposed scenarios, the nanoplatelets themselves can initiate the formation of the disclination lines and their self‐assembly.

Also the high affinity of nanoparticles to the disclination lines can be responsible for the formation of the magnetic nanochains which turn into entangled 3D networks with loops and knots.

The external magnetic field brings about the restructuring of the network observed in the experiment. The coupling between the magnetization and the nematic director controls the polar state, resulting in the magnetoelectric effect. To sustain the remanent magnetization, the topology of the network should be compatible with the vectorial symmetry. Since the orientation of the lines is given by the 1D magnetic order of the nanoplatelets, the remanent magnetization will be sustained for the lines pinned at the substrate or locked by the neighboring knots as suggested in the scheme Figure 6e–g.

The switching occurs via magnetic inversion within the disclination lines (Figure 6a) or displacement of bifurcation points (Figure 6b–d). The inversion can be realized through the propagation of the soliton‐like structures along the disclination lines. Such propagating fronts can be seen as disturbances in the disclinations observed in thin cells in polarizing microscopy.

In case of the looped knots, inversion walls of the magnetization within the disclinations can stabilize the magnetized state. Branched structures with mobile links attached to the inversion walls can determine the switching mechanism allowing the magnetization switch between the two magnetized states (Figure 6e–g). This explains why exposure to magnetic fields (>0.1 T) is required to obtain the magnetized state. The magnetically mediated (viscous) mechanoelectric effects are responsible for the electric response because it has been shown that the viscous mechanoelectric response exists in the pure ferroelectric nematic system.^[^ ^24^ ^]^ The magnetized domain walls/disclinations that separate the ferroelectric domains rearrange, leading to a transient magnetoelectric response. Although the material is locally ferroelectric, as demonstrated by nonlinear optics, understanding the mechanism of the polarization‐magnetization coupling on the global scale (in bulk) remains challenging. Studies of colloidal knots in nematics showed that they generate topologically non‐trivial structures of topological defects such as boojums resulting in elastic couplings between the particles and the defects.^[^ ^47^ ^]^ The director, confined in such a region is fixed by the topological constraints and can be controlled via alignment of the defects. Thus, the reorganization of the links in the network allows the realignment of the polarized domains within the network in a polar fashion.

High degree of disorder in the magnetic network leads to the reduction of the electrically induced magnetic response compared to the magnetically‐induced electric response. The results shown here open a promising route for the development of soft multiferroic systems. The coupling between the magnetic and electric order types can be improved by either tailored functionalization of the nanoplatelets or designing asymmetric Janus‐type platelets.

These room‐temperature soft multiferroics, exhibiting coupled magnetic and electric order, hold promise not only for advancing fundamental science but also for their strong applicative potential, creating opportunities for soft and flexible sensors and multi‐stimuli responsive actuators in soft robotics and wearable technology.

## 4 Experimental Section

The multiferroic suspension was prepared by mixing an adequate amount of scandium‐doped barium hexaferrite suspension with M5 ferroelectric liquid crystal mixture (Merck). The mixture was heated to 100° to fully evaporate the carrier fluid 1‐butanol and quenched on top of neodymium magnets (482 mT) to room temperature. The BaHF suspension was prepared as described in refs. [46, 48]. The selected composition range reflects our previous work on ferromagnetic liquid crystals, which was conducted between 0.3 wt% and 3 wt%. Above 3 wt%, nanoparticles aggregate, necessitating centrifugation and leaving the effective particle concentration in the suspension uncertain.

In the multiferroic suspensions, a detectable response was observed even at 0.3 wt%; however, this concentration proved too low for reliable signal detection in reverse magnetoelectric measurements. Interestingly, the maximum nanoparticle concentration achievable without obvious aggregation is higher in the multiferroic suspensions (4 wt%) than in the purely ferromagnetic liquid crystal system (3 wt%). Yet during the course of our experiments, the 4 wt% multiferroic suspension aged gradually and formed aggregates–an effect we attribute to confinement, since bulk suspensions of identical composition remained stable.

To characterize the optical anisotropy of the liquid crystal, polarization microscopy studies were made using AxioImager A.1 polarizing microscope (Carl Zeiss GmbH, Germany) equipped with a heat stage (Instec, USA). Samples were prepared in commercial glass cells (E.H.C., Japan and WAT, Poland) with planar transparent indium tin oxide (ITO) electrodes (cell thickness: 2.5, 3, 5, 10, 25 µm, ITO resistance: 10 Ω). The cells were filled with the hybrid LC by capillary forces in the isotropic state and quenched to room temperature in a magnetic field of 140 mT. Cells with bare ITO/glass substrate were used as well as treated cells with alignment layers. Cells with thickness 2.5 µm were preferred to suppress the disordered texture and spontaneous formation of twisting of the director. The selection of cell thickness and surface treatment was guided by the balance between surface anchoring and bulk elastic forces in the nematic phase. In ferroelectric nematics, spontaneous ambidextrous twist often arises, which can be effectively suppressed in cells thinner than 5 µm. Moreover, the optically unresolved disclination disorder is minimized in such thin films, approximating a quasi‐2D state. This optical clarity was best achieved using 2.5 µm thick cells. Thicker cells, although more prone to director disorder, were used where higher signal strength (magnetization, current transient) was required. The aligning layers used are rubbed polyimide layers for LC planar alignment (parallel and antiparallel rubbing), and cetyltrimethylammonium bromide for the vertical alignment. The vertical alignment, however, could be achieved only in the non‐polar nematic phase. Cells with interdigitated in‐plane electrodes (IPS) were used to study in‐plane switching (INSTEC, USA).

The structure of the director field and the polarization was investigated using polarizing confocal laser scanning microscopy (Leica TCS SP8, CLSM). Generation of the optical second harmonic (SHG) was measured using the multiphoton laser of the confocal microscope. A tunable IR laser (λ_ex_ = 880 nm) was used as a fundamental light beam. The direction of maximal SHG efficiency was mapped in the SHG microscopy images to indicate the local direction of the polar axis. This is possible since the nonlinear coefficient *d*_33_ ≫ *d*_31_.^[^ ^23^ ^]^

The complex magnetic susceptibility was measured with a commercially available AC susceptometer (Dynomag, RISE Acreo, Gothenburg, Sweden) with an excitation flux of 0.5 mT in a frequency range of 1 Hz to 500 kHz. The samples were measured in cylindrical vials containing 100 µL of the hybrid‐LC.

The optical transmission of the sample cells between crossed polarizers were carried out in a custom‐made setup containing tungsten and laser sources, coil system, photodetector, CCD camera and a long‐range microscope. The sample was placed in an oscillating magnetic field of a four‐coil system, allowing arbitrary variation of the exciting field angle with respect to the position and/or the rubbing direction of the cell. Transmission was characterized using a cw He‐Ne laser source λ = 632 nm, *P* = 10 mW (JDS Uniphase) and the transmitted polarized light was detected by a photodetector (Thorlabs PDA36A2, detection range between 350 and 1100 nm). The measurement was performed in the frequency range of the magnetic field from 0.1 Hz to 100 Hz. The phase shift between the applied field and the transmission signal was determined from the analysis of the time series.

Magnetization measurements were made using a fluxgate magnetometer sensor AS‐UAP GEO‐X Projekt Elektronik GmBH (Germany) and SQUID MPMS‐3 (Quantum Design Europe). SQUID measurements were performed on samples filled in 20 mm long thin capillaries with a diameter of 1 mm.

The transient currents in the magnetic field across the LC cell were measured using a picoammeter (Keithley 6487) with an accuracy 100 fA. The picoammeter was connected to a digital oscilloscope PicoScope 6480. The cells were magnetized using a 640 mT electromagnet. The current transient was recorded on switching on the field (640 mT) of the opposite polarity (scheme in Figure 5d). A system of electromagnets was used with a pole diameter of 90 mm and the field uniformity of 0.3%. In case of electric switching, we used cells with interdigitated comb electrodes (INSTEC) and recorded current transient across a 4 kΩ resistor using a PicoScope 6400 digital oscilloscope.

## Conflict of Interest

The authors declare no conflict of interest.

## Supporting information

Supporting Information

Supporting Information

Supplemental Movie 1

Supplemental Movie 2

Supplemental Movie 3

Supplemental Movie 4

Supplemental Movie 5

Supplemental Movie 6

## Data Availability

The data that support the findings of this study are available from the corresponding author upon reasonable request.
