# Supplementary material for: Room‐Temperature Multiferroic Liquids: Ferroelectric and Ferromagnetic Order in a Hybrid Nanoparticle–Liquid Crystal System
Source: Adv Mater. 2025 Jul 26;37(41):e08406. doi: 10.1002/adma.202508406 (PMC12531723; doi:10.1002/adma.202508406)
Supplement: Supplementary file 1 — Supporting Information [file ADMA-37-e08406-s001.pdf]

ADVANCED MATERIALS

Supporting Information

for *Adv. Mater.*, DOI 10.1002/adma.202508406

Room-Temperature Multiferroic Liquids: Ferroelectric and Ferromagnetic Order in a Hybrid Nanoparticle–Liquid Crystal System

Hajnalka Nádasi, Peter Medle Rupnik, Melvin Küster, Alexander Jarosik, Rachel Tuffin, Matthias Bremer, Melanie Klasen-Memmer, Darja Lisjak, Nerea Sebastián, Alenka Mertelj, Frank Ludwig and Alexey Eremin*

Supporting Information for

Room-Temperature Multiferroic Liquids: Ferroelectric and Ferromagnetic Order in a Hybrid Nanoparticle-Liquid Crystal System

Hajnalka Nádasi Peter Medle Rupnik Melvin Küster Alexander Jarosik
Rachel Tuffin Matthias Bremer Melanie Klasen-Memmer
Darja Lisjak Nerea Sebastián Alenka Mertelj Frank Ludwig Alexey Eremin

Supplementary Note 1. Abbreviations

BaHF	barium hexaferrite
ITO	indium tin oxide
LC	liquid crystal
N	nematic phase
N_F	ferroelectric nematic phase
M	M-phase
POM	polarising optical microscopy
SHG	optical second harmonic generation

Supplementary Note 2. Electric and magnetic behaviour

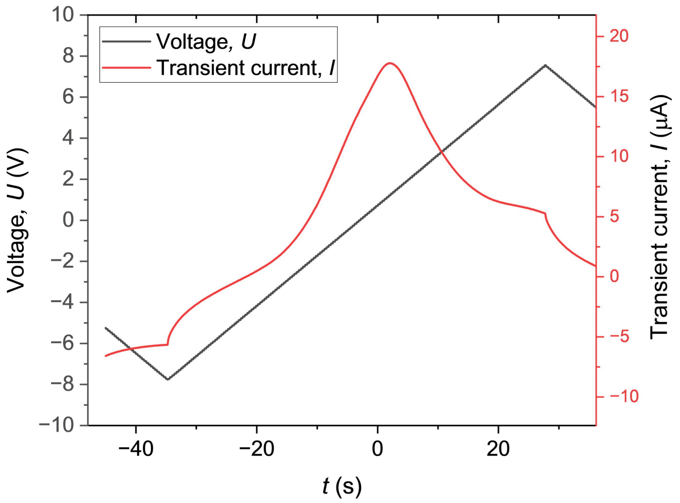

Figure S1: **Current transient** in response in the LC-hybrid system (3 wt%) to the triangular-wave voltage measured in a 6 µm thin cell with polyimide aligning layers and in-plane electrodes ($T = 21\,°C$, inter-electrode distance 20 µm).

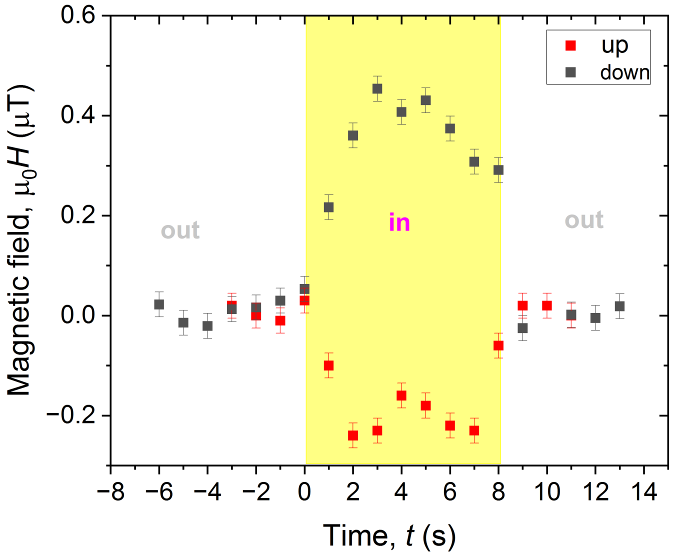

Figure S2: **Remanent magnetisation in a thin cell:** Magnetic field measured at the surface of the 10 µm thick V-type cell inserted under a fluxgate sensor. The cell was prepared by quenching in 700 mT field applied upwards (dataset "up") and downwards (dataset "down"). The measurements were done in a magnetically screened chamber.

Supplementary Note 3. Optical behaviour

Polarising microscopy textures provide information on the molecular order and orientation in the liquid crystal phase. In uniformly aligned thin nematic layers, the apparent colour is determined by the effective birefringence of the sample and the optical extinction direction is determined by the orientation of the optical axis. Thick cells as in Fig S3a show a disordered texture consisting of thin lines. The same texture remains in the M phase of the hybrid material (Fig S3b). However, in the latter case, no reaction to applied electric or magnetic fields could be found.
The optical transmittance of a thin layer uniformly planar-aligned nematics exhibits four extinctions and four maxima upon the 360° sample rotation. Twisted director structures can be determined through their optical rotation using slightly uncrossed polarisers or observing the textures in circularly polarised light.
The ferroelectric character of the molecular order in the N_F-hybrid is manifested in the current transients in response to the applied triangular-wave voltage between the electrodes. The current peak is produced by the polarisation reversal and is similar to that measured in the N_F of the pure compound (Fig. S1).
In thin (2.5 µm) cells with antiparallel rubbed polyimide aligning layers, the nematic director favours twisted configuration in the N_F phase. However, occasionally, uniformly aligned domains occur too. In case of the hybrid N_F, stripped domains (Type 1) with the preferred orientation along the director are observed as well as the twisted domains (Type 2) (Fig S4a,b). The domains can also be distinguished by their optical transmittance as a function of the angle φ between the polariser and the cell's aligning direction. Compared to Type 1 domains, the Type 2 domains show much smaller difference between the transmission maxima and minima (Fig S4c).
At a high magnification, the striped domains additionally display a splay deformation of the director. This can be seen from the displacement of the extinction brushes upon rotating the sample between crossed polarisers (Fig S3a-c).

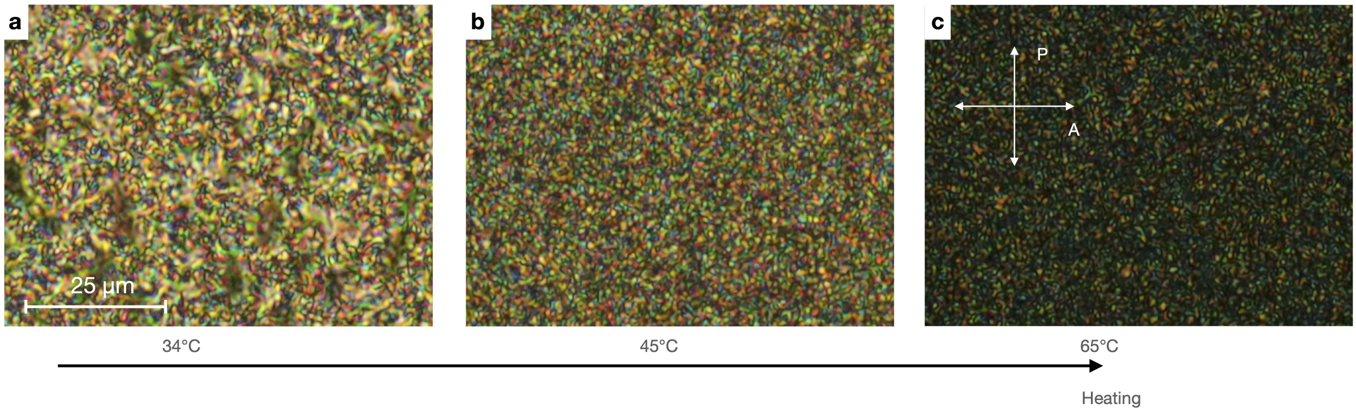

Figure S3: **Temperature-driven transformations of polarising microscopy textures in hybrid-LC:** on heating in 10 µm cell with polyimide rubbed substrates in **a** the hybrid N_F phase at $T = 34\,°C$, **b** M phase $T = 45\,°C$, and **c** in the N phase $T = 65\,°C$.

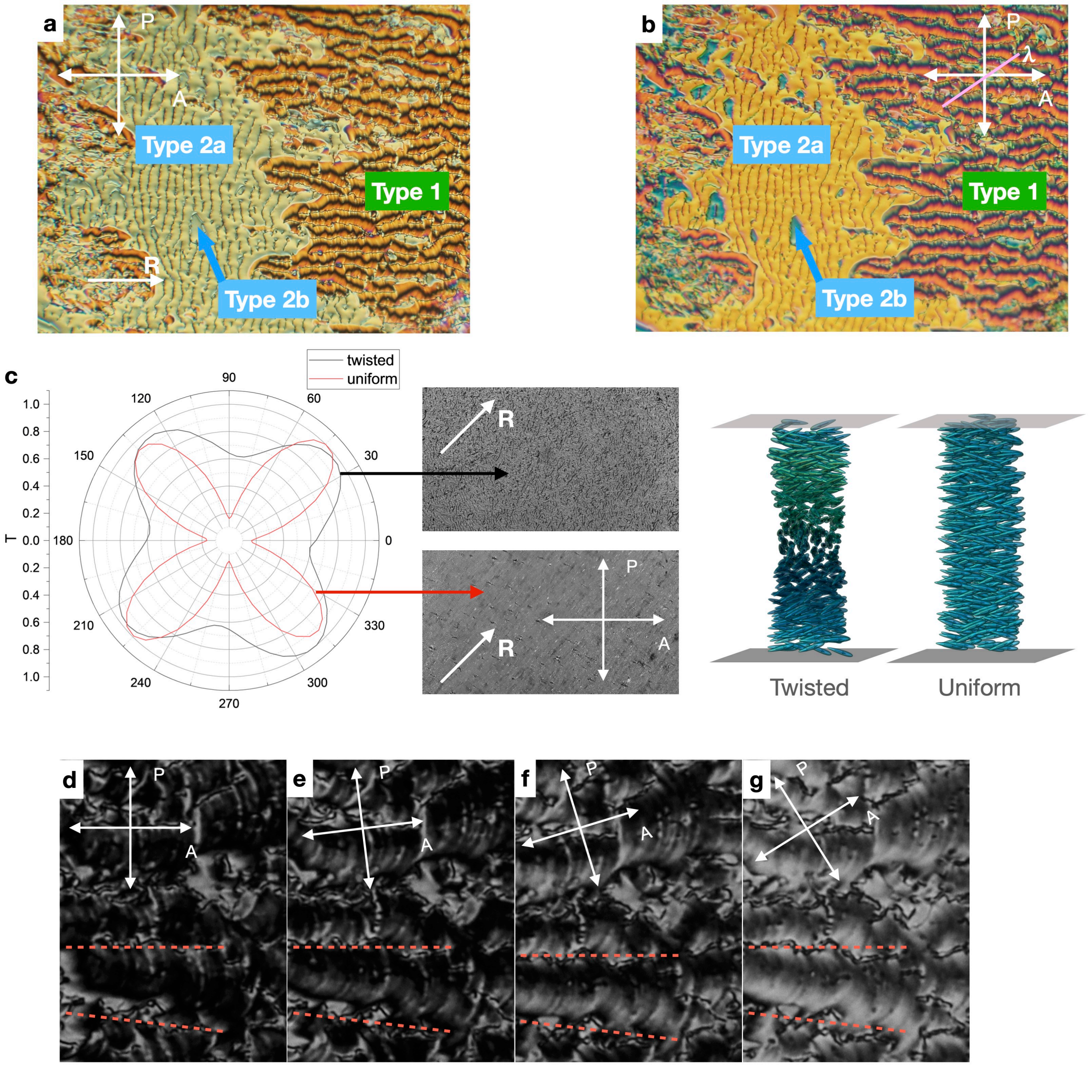

Figure S4: **Optical domain structures in thin cells: a** POM image of the N_F texture in a 2.5 µm P-type cell between crossed polarisers and **b** with a full-wave plate inserted. **c** Normalised transmittance in the twisted and uniform stripped domains as a function of the angle between the polariser and the cell's aligning layer direction **R** with corresponding optical textures. On the right side displays schematics of the twisted and uniformly aligned nematic structures, corresponsingly. **d - g** Director stripes in Type 1 domains depicted at different angles between the aligning layer direction an the polarisers. The shift of the extinction brush suggests a splay deformation of the director within the stripe.

Supplementary Note 4. Morphology of nanoplatelet/N_F hybrid confined to 10 µm V-cell.

In the V-cells, the aligning layer favouring orthogonal alignment of conventional nematic phases is not sufficient to achieve the orthogonal alignment in the N_F phase. For the morphological studies isotropic 0.3 wt% nanoplatelet/N_F hybrid-LC was filled into V-cells and quenched to the N_F phase in 0.5 T homogenous magnetic field perpendicular to the substrate surface. The nanoplatelet/N_F hybrids exhibit even more complex and various morphologies resulted from the competition between the aligning interactions, bulk elasticity and the distortions introduced by the nanoparticles. Large disordered (Fig. S5a), grainy (Fig. S5b), striped (Fig. S5c) and uniform domains (Fig. S5d,e) were observed. Similarly to the P-cells, the grainy texture consists of disclination lines which are responsive to magnetic fields in the range of 100 mT. Some domains, however, did not show any magnetic response as in (Fig. S5a, b) suggesting assembly of the nanoplatelets in the non-magnetic (antiparallel) fashion.

Magnetically responsive regions with uniform patches on a 20 µm scale were formed typically outside the ITO-electrode area of the cell. Such regions were not observed in P-cells. In these disclination-free areas of Fig. S5d repeated application of a horizontal magnetic field of opposite signs alters the transmitted light intensity (I) and shifts the domain borders (Fig. S5e). After the removal of the magnetic field, the texture relaxes to a state different from the initial one. To explore the effect of the horizontal magnetic field, I was measured in several regions of different domains at different orientation of the crossed polarisers. Fig. S6 shows the intensity profiles with and without magnetic field. The results show that without magnetic field (Fig. S6a) director $\mathbf{n}$ varies domain by domain. However, application of the magnetic field (Fig. S6b) has an ordering effect, since most of the intensity profiles almost coincide with intensity maxima at 0° and 90°, i.e. when the external field is aligned along one of the polarisers.

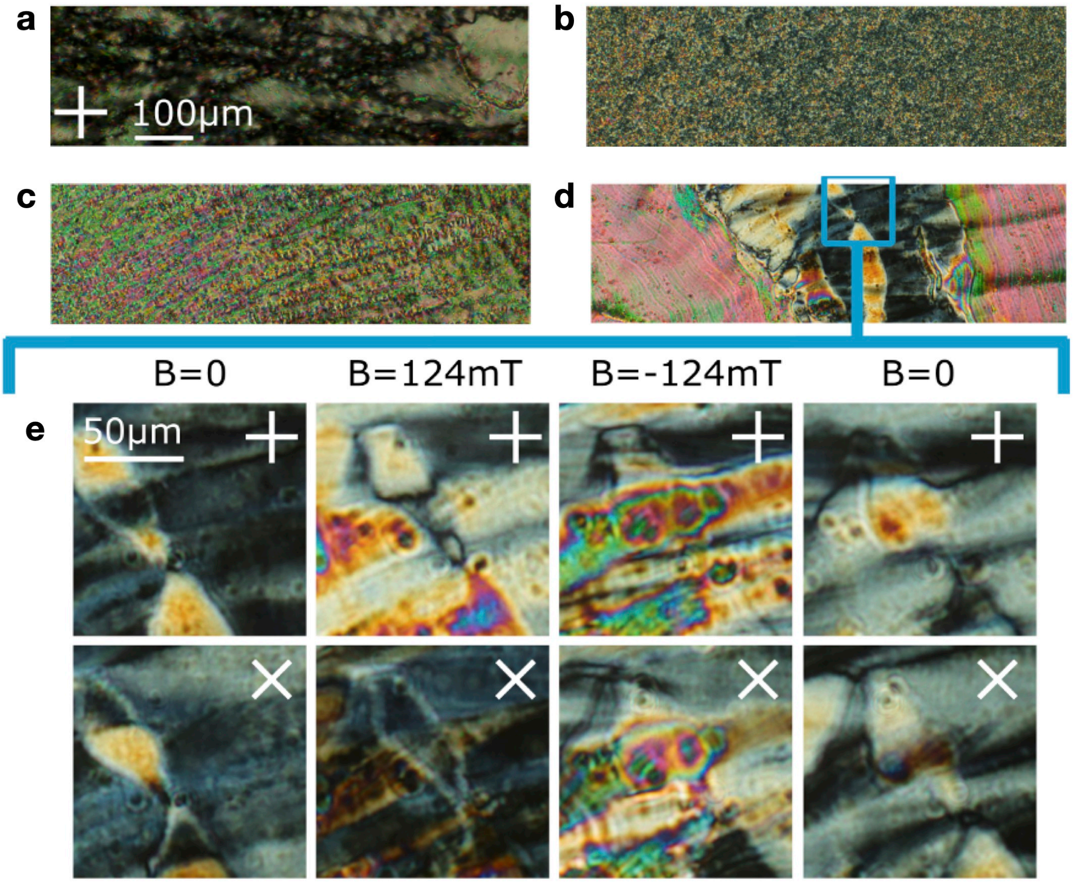

Figure S5: **Morphological observations of nanoplatelet/N_F hybrid confined to 10 μm V-cell using POM.** **a** Disordered magnetically irresponsive texture. **b** Magnetically irresponsive grainy texture. **c** Magnetically responsive stripped texture. **d** Magnetically responsive uniform texture. **e** Magnetooptical response in the region marked by a blue rectangle in **d** The images are taken with crossed polarisers at the magnetic field strengths marked above images. In the upper row, the polariser is aligned horizontally, and in the bottom row, it is rotated by 45° (white marks show the polariser's alignment).

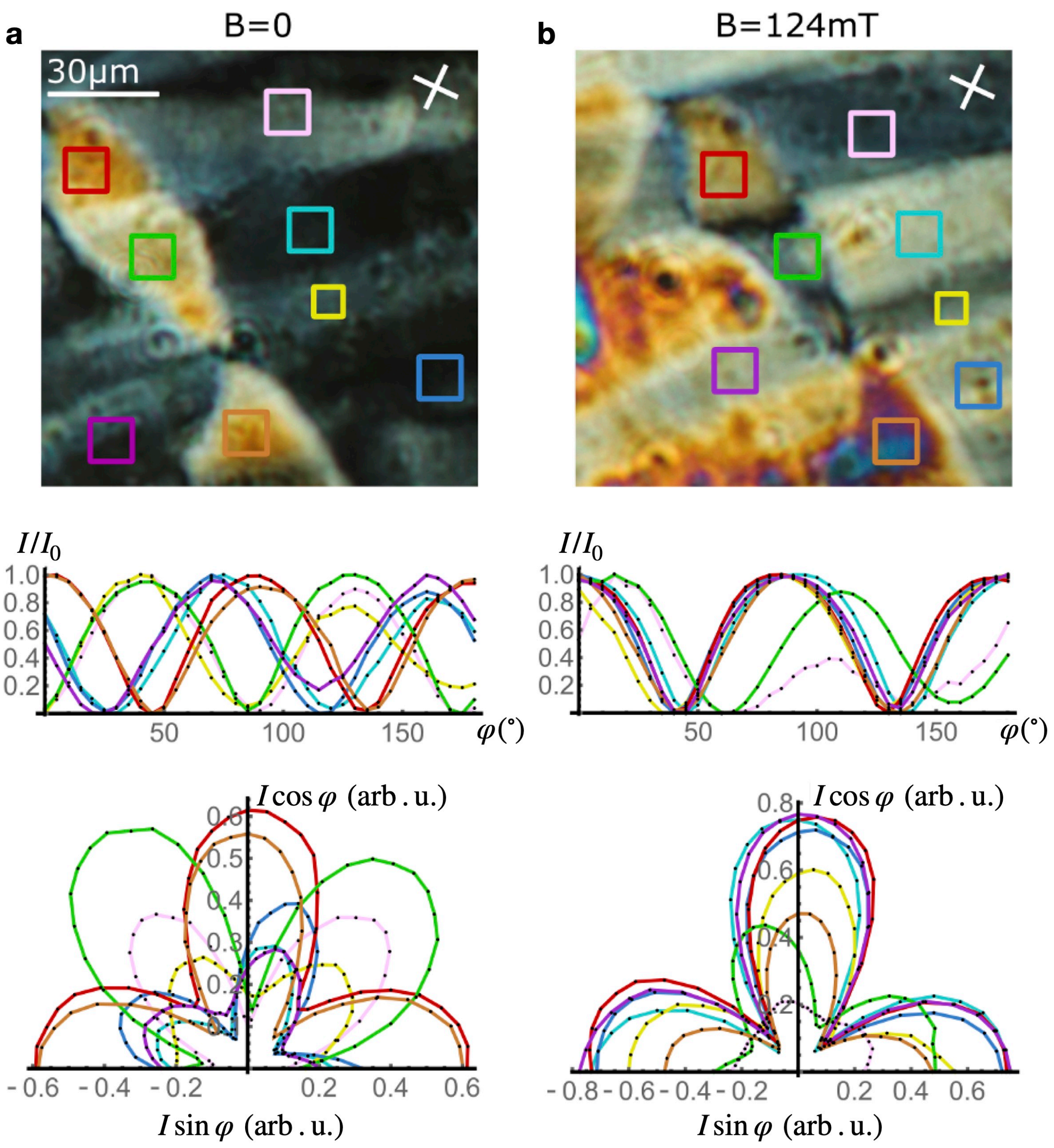

Figure S6: **Polarising microscopy analysis of the polydomain structure of nanoplatelet/$\mathrm{N_F}$ hybrid confined to a 10 µm V-cell.** Results shown in Figure S5d are here further analysed for the cases **a** without external magnetic field and **b** when the in-plane magnetic field was applied. Eight different domains were analysed each denoted by a distinct color. The plots below the microscopic images show the normalised intensity of transmitted light depending on the orientation of the polarisers. The bottom parametric plots display the non-normalised intensity of transmitted light (average value of the red, blue, and green pixels in the photograph) for different angles. Photographs were taken as the sample was rotated with respect to the crossed polarisers. The external magnetic field was always applied in the same direction with respect to the sample. Images **a** and **b** were taken for the sample aligned at 115°. The borders between different domains were clearly visible due to significant changes in the transmitted light intensity.

Supplementary Note 5. Optical response of Type 1 domain of nanoplatelet/$\mathrm{N_F}$ hybrid confined to 2.5 µm P-cell to external magnetic field.

In the Type 1 domains of nanoplatelet/$\mathrm{N_F}$ hybrid confined to 2.5 µm P-cell the external magnetic field disturbs the disclination lines and causes buckling instability as observed by POM in Figure 6a. The disclination lines are coupled to the electric polarisation orientation of the surrounding $\mathrm{N_F}$ phase, so their magnetically induced movement causes rearrangement of the polarisation structure

within the sample. This is shown in Figure S7 where SHG signal is recorded in dependence on the polarisation orientation of the incoming probing IR beam. Second order dielectric susceptibility tensor d_{ij} for this class of compounds satisfies the conditions for its components $d_{33} > d_{31}$, where the 33 axis is along the nematic director. This allows determining the local polar axis alignment from the direction of maximal SHG efficiency. (see Folcia CL, Ortega J, Vidal R, Sierra T, Etxebarria J. The ferroelectric nematic phase: an optimum liquid crystal candidate for nonlinear optics. Liq Cryst. 2022:1-8. doi:10.1080/02678292.2022.2056927 and Sebastián N, Lovšin M, Berteloot B, et al. Polarization patterning in ferroelectric nematic liquids via flexoelectric coupling. Nat Commun. 2023;14(1):3029. doi:10.1038/s41467-023-38749-2)

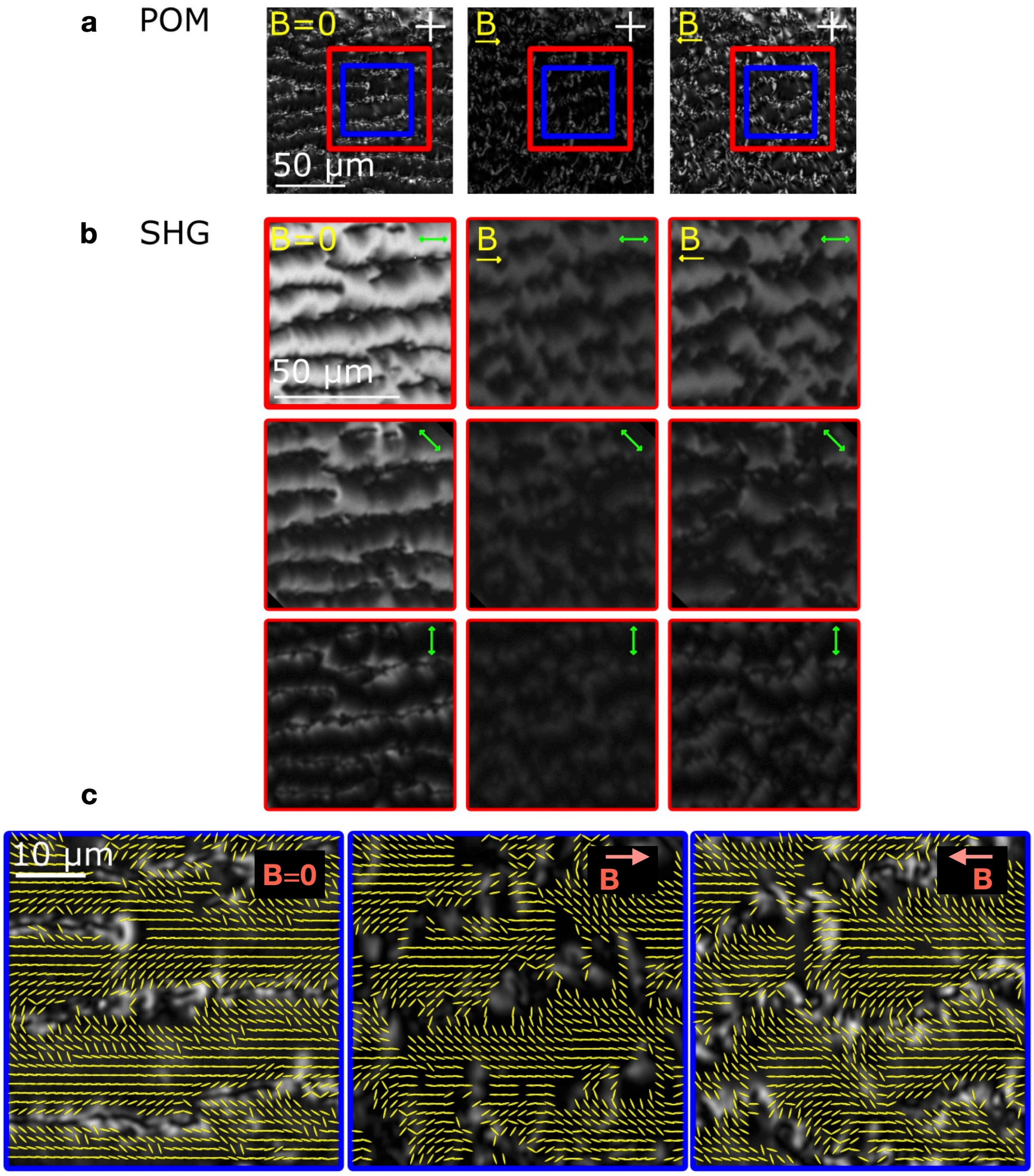

Figure S7: **POM and SHG analysis of Type 1 domains of nanoplatelet/N$_F$ hybrid confined to 2.5 µm P-cell.** **a** POM images correspond to the cases without magnetic field and with applied magnetic fields of opposite polarities. Part **b** shows the SHG microscopy of the corresponding regions in **a** marked by red rectangles. The images were taken at three different orientations of polarisation of the incoming IR beam (green arrows) without an analyser. The figures show how the external magnetic field affects the SHG efficiency. Analysis of the SHG microscopy images allows us to extract the direction of the highest efficiency corresponding to the direction of the local polarisation. In **c** the polarisation field marked in yellow is overlain with the POM images marked blue in **a**.

Supplementary movie 1

Bistable magnetooptical response of a network texture in a 6 µm thick cell treated for vertical alignment. The image width is 6 µm, crossed polarisers are aligned along the image sides.

Supplementary movie 2

Rearrangement of the disclination network in response to an external magnetic field observed in a 5 µm cell with a bare glass substrate (no aligning treatment). The image width is 250 µm, crossed polarisers are aligned along the image sides.

Supplementary movie 3

SHG microscopy on a 5 µm cell exposed to an external magnetic field for Fig. 2f.

Supplementary movie 4

Field-induced rearrangement of the disclination lines in striped domains aligned along the alignment direction a 3 µm cell with a planar alignment. The image width is 190 µm, crossed polarisers are aligned along the image sides.

Supplementary movie 5

Soliton-like distortion propagation along the disclination lines in the Type 1 domains observed in a 2 µm thin cell with antiparallel planar surface treatment. The image width is 205 µm, crossed polarisers are aligned along the image sides.

Supplementary movie 6

Switching in twisted domains in a 3 µm thick cell with polyimide rubbing ($c = 0.3$ wt%). The image width is 225 µm, crossed polarisers are aligned along the sides of the image.

Supplementary movie 7

Propagation of the director distortion along a disclination line after poling the magnetic field ($B = 140\,\mathrm{mT}$, the image height is 12 µm, cell thickness $d = 2.5\,\mu\mathrm{m}$
