# Supplementary figures and images for: Room‐Temperature Multiferroic Liquids: Ferroelectric and Ferromagnetic Order in a Hybrid Nanoparticle–Liquid Crystal System
Source: Adv Mater. 2025 Jul 26;37(41):e08406. doi: 10.1002/adma.202508406 (PMC12531723; doi:10.1002/adma.202508406)

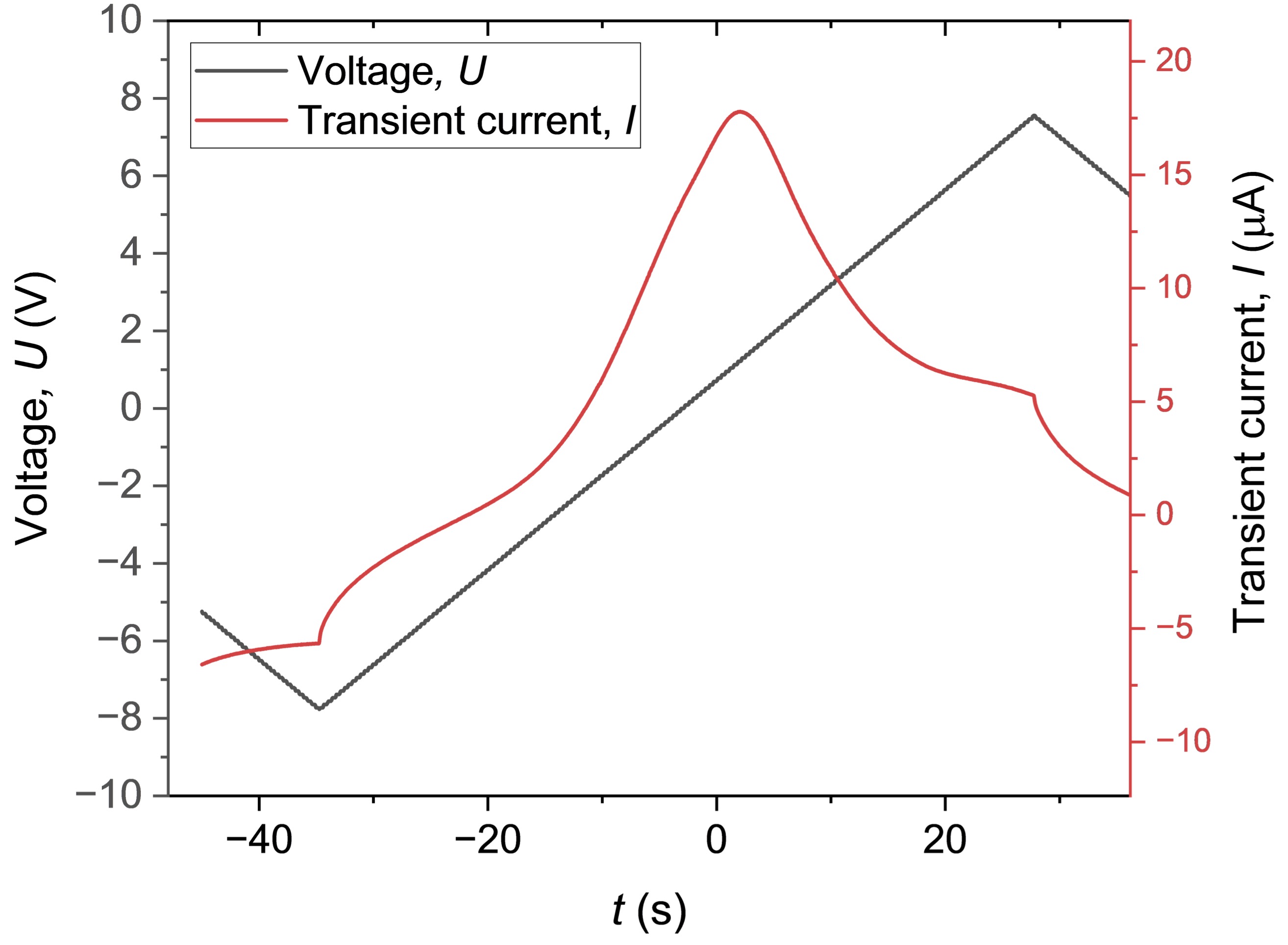

Supplement: Supplementary file 2 — Supporting Information [file ADMA-37-e08406-s008.zip › fig_S1.jpg]

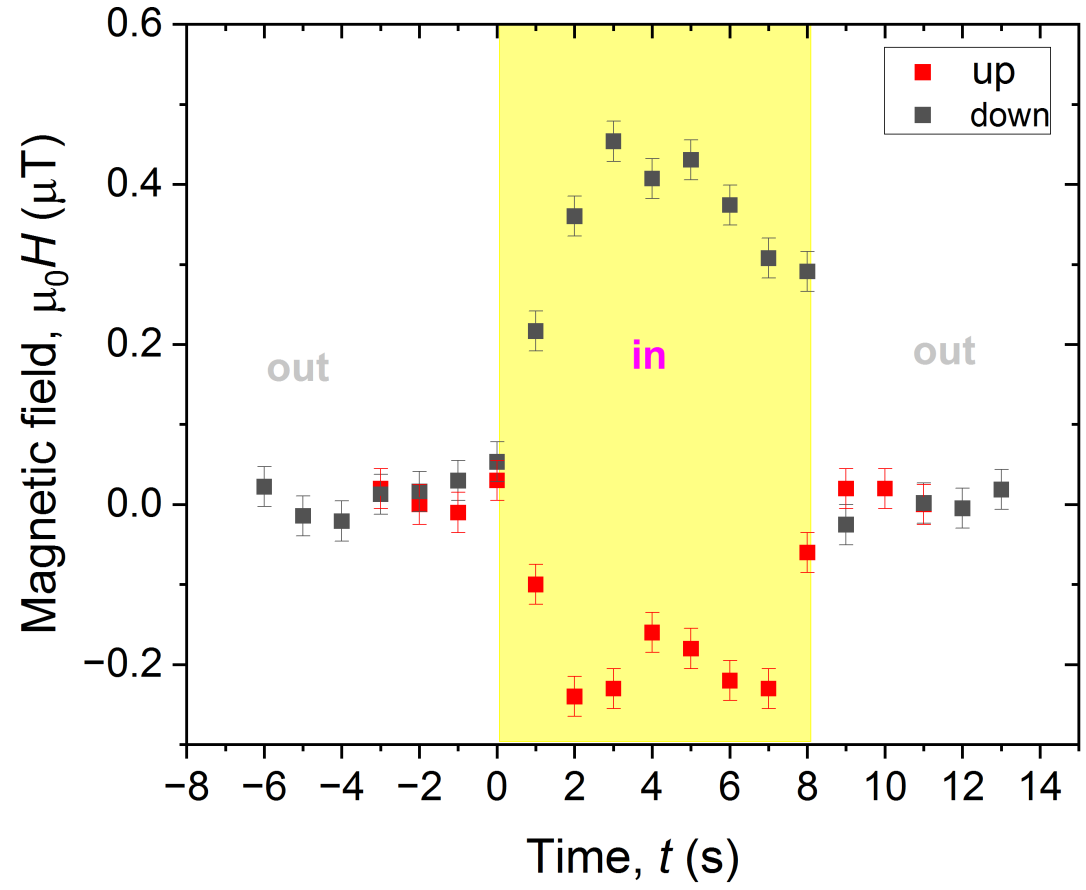

Supplement: Supplementary file 2 — Supporting Information [file ADMA-37-e08406-s008.zip › fig_S2.pdf]

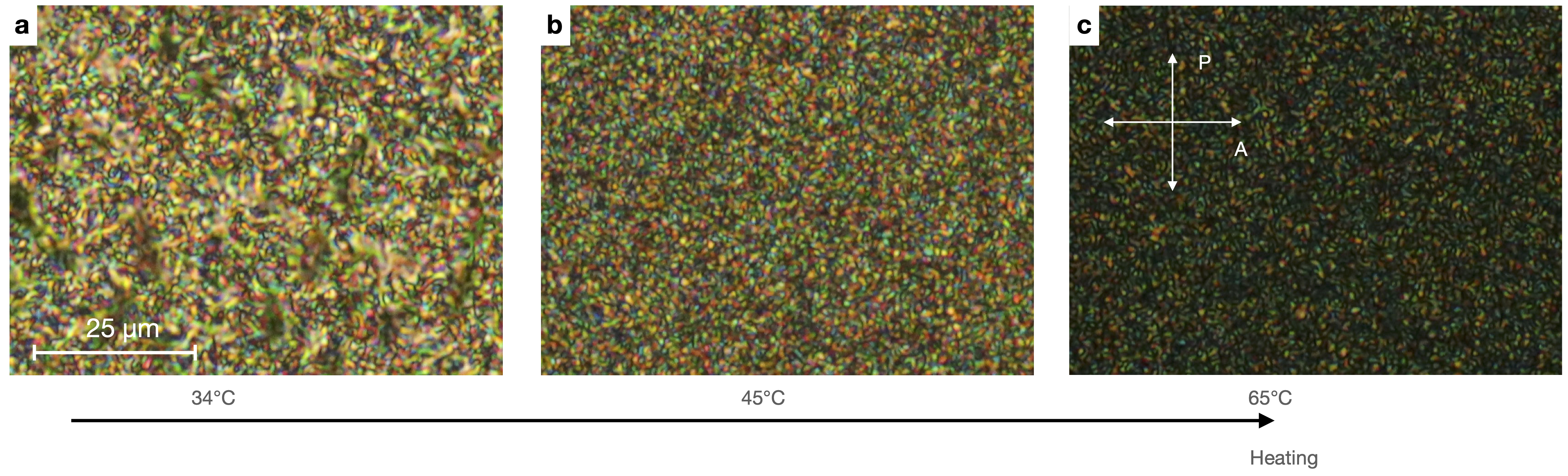

Supplement: Supplementary file 2 — Supporting Information [file ADMA-37-e08406-s008.zip › fig_S3.png]

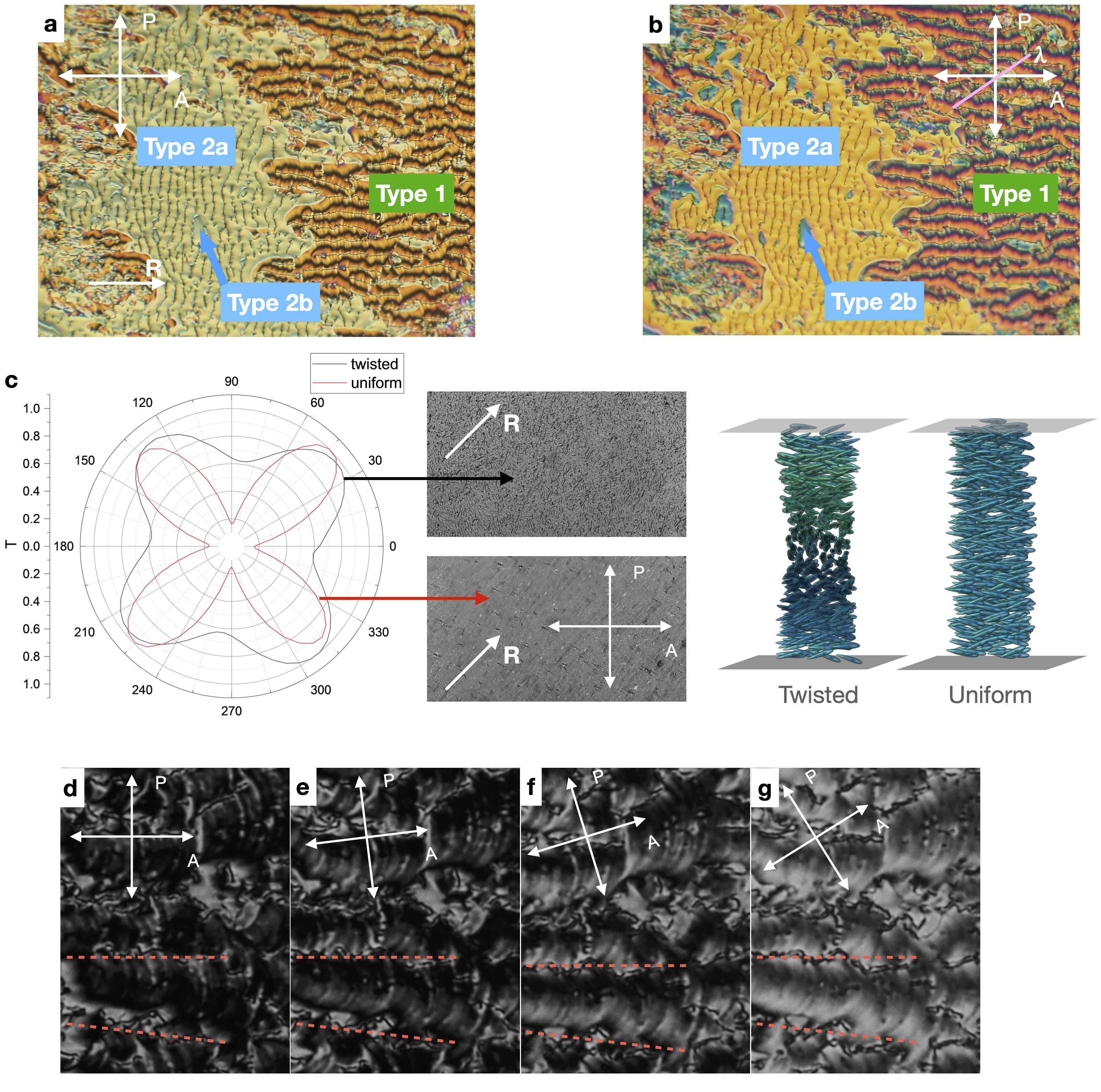

Supplement: Supplementary file 2 — Supporting Information [file ADMA-37-e08406-s008.zip › fig_S4.jpg]

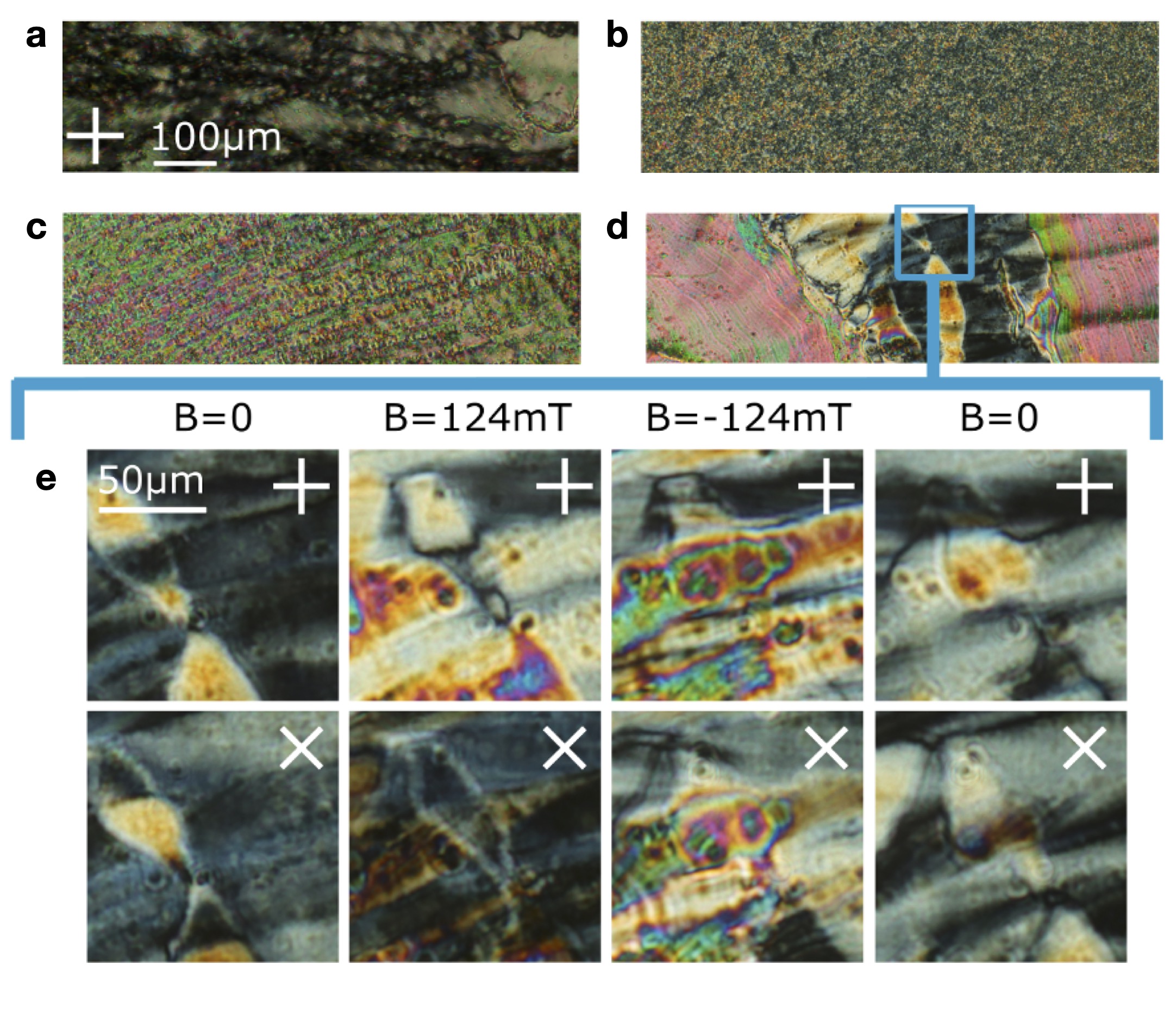

Supplement: Supplementary file 2 — Supporting Information [file ADMA-37-e08406-s008.zip › fig_S5.jpg]

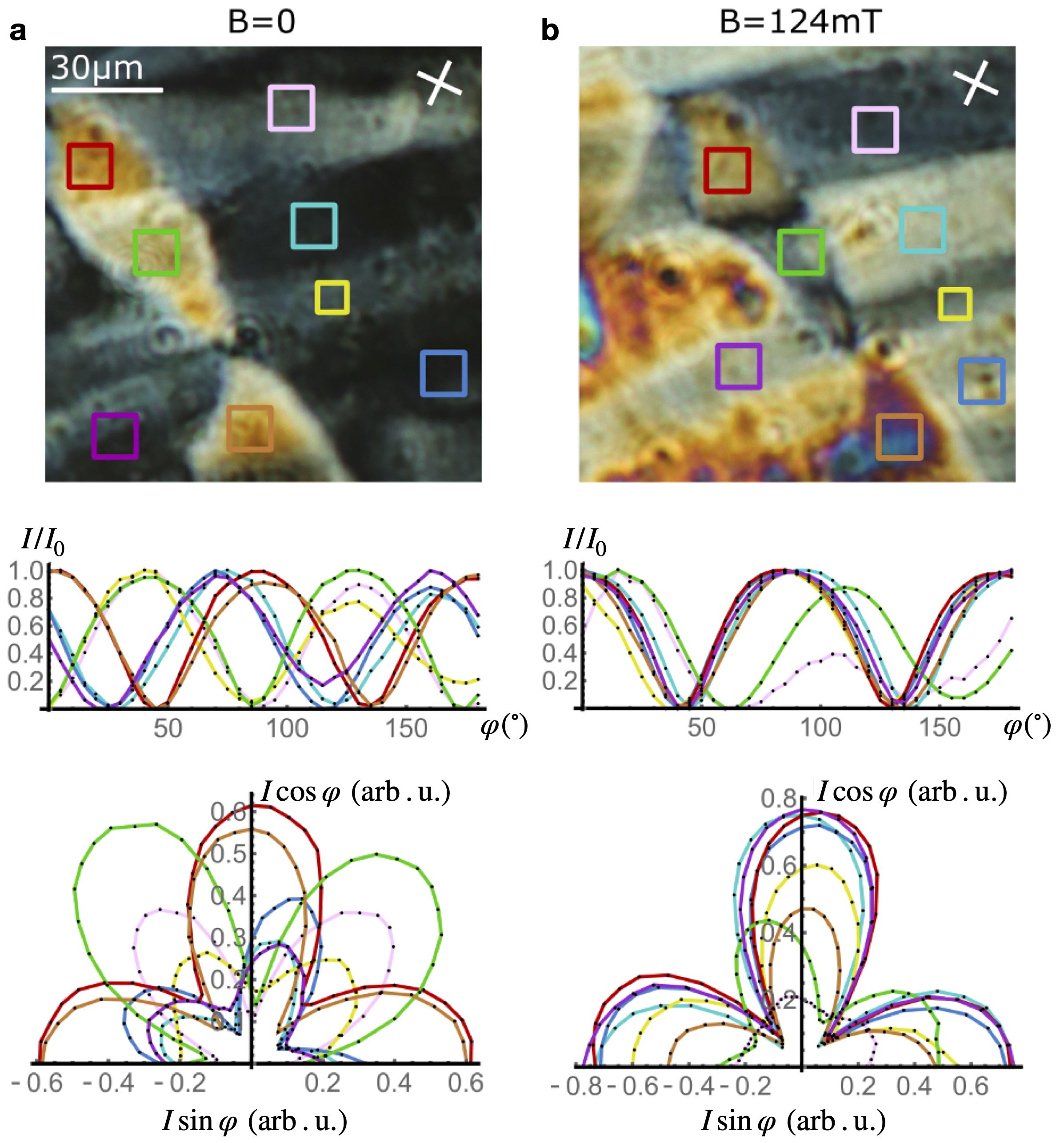

Supplement: Supplementary file 2 — Supporting Information [file ADMA-37-e08406-s008.zip › fig_S6.jpg]

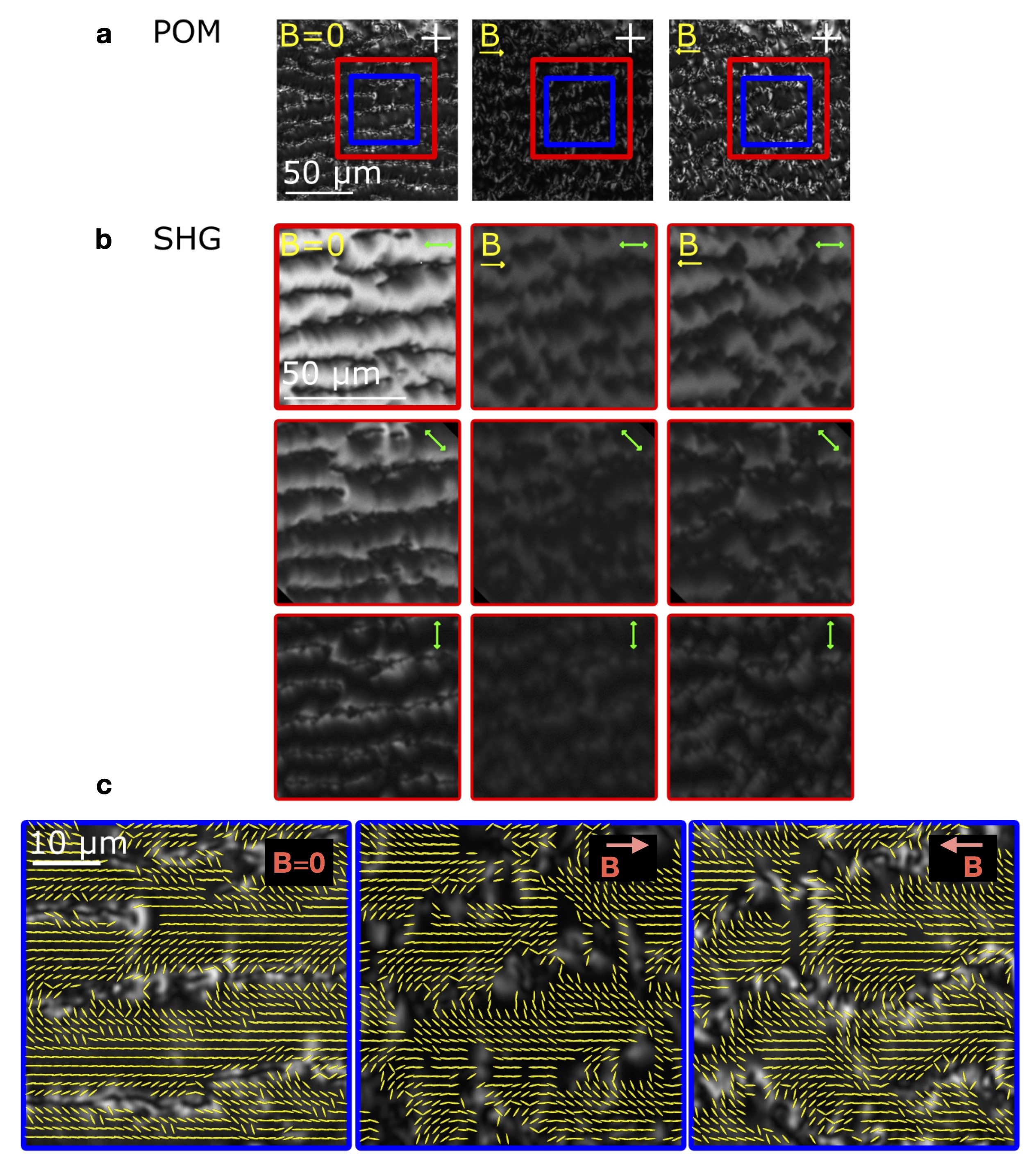

Supplement: Supplementary file 2 — Supporting Information [file ADMA-37-e08406-s008.zip › fig_S7.jpg]
